# Three Polyborates with High-Symmetry [B_12_O_24_] Units Featuring Different Dimensions of Anion Groups

**DOI:** 10.1021/acsomega.3c02248

**Published:** 2023-06-02

**Authors:** Yu Dang, Jingdong Yan, Xueling Hou, Hongsheng Shi

**Affiliations:** † Research Center for Crystal Materials, CAS Key Laboratory of Functional Materials and Devices for Special Environments, Xinjiang Key Laboratory of Electronic Information Materials and Devices, Xinjiang Technical Institute of Physics & Chemistry, CAS, 40-1 South Beijing Road, Urumqi 830011, China; ‡ Center of Materials Science and Optoelectronics Engineering, University of Chinese Academy of Sciences, Beijing 100049, China

## Abstract

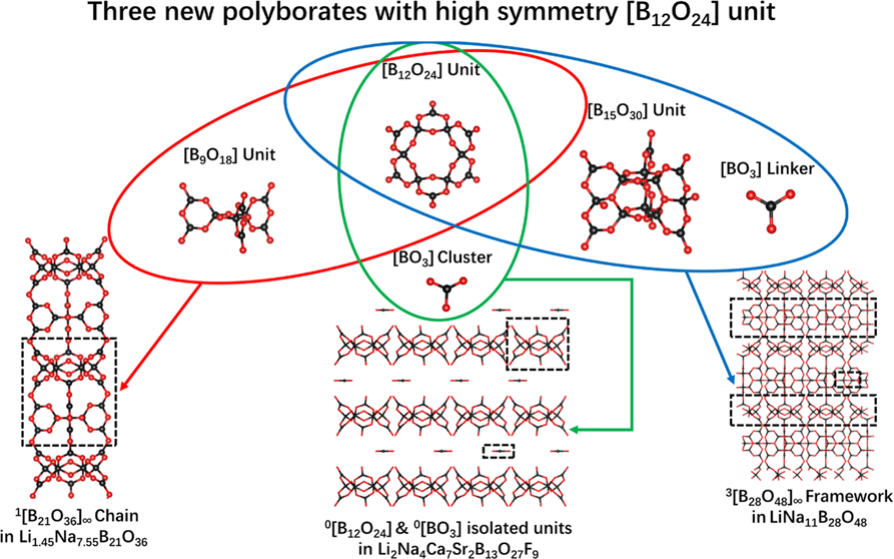

Three polyborates, namely, LiNa_11_B_28_O_48_, Li_1.45_Na_7.55_B_21_O_36_, and Li_2_Na_4_Ca_7_Sr_2_B_13_O_27_F_9_, were synthesized via the high-temperature solution method. All of them feature high-symmetry [B_12_O_24_] units, yet their anion groups exhibit distinct dimensions. LiNa_11_B_28_O_48_ features a three-dimensional anionic structure of ^3^[B_28_O_48_]_∞_ framework, which is composed of three units: [B_12_O_24_], [B_15_O_30_], and [BO_3_]. Li_1.45_Na_7.55_B_21_O_36_ possesses a one-dimensional anionic structure of ^1^[B_21_O_36_]_∞_ chain consisting of [B_12_O_24_] and [B_9_O_18_] units. The anionic structure of Li_2_Na_4_Ca_7_Sr_2_B_13_O_27_F_9_ is composed of two zero-dimensional isolated units, namely, [B_12_O_24_] and [BO_3_]. The novel FBBs [B_15_O_30_] and [B_21_O_39_] are present in LiNa_11_B_28_O_48_ and Li_1.45_Na_7.55_B_21_O_36_, respectively. The anionic groups in these compounds exhibit a high degree of polymerization, thereby augmenting the structural diversity of borates. And the crystal structure, synthesis, thermal stability, and optical properties were meticulously discussed to guide the synthesis and characterization of novel polyborates.

## Introduction

Borates are extensively utilized in the fields of photoelectric functional materials, glass materials, ceramic materials, fire-resistant materials, and additive materials owing to their diverse structures and exceptional properties.^1,2^ Among them, photoelectric functional materials play a crucial role in various advanced optical devices such as lasers, polarizers, optical isolators, and photolithography. They have extensive applications in scientific research, military technology, medical treatment, and other fields. In particular, several borates such as BaB_2_O_4_ (BBO),^3^ LiB_3_O_5_ (LBO),^4^ KBe_2_BO_3_F_2_ (KBBF),^5^ and others have garnered significant attention in the field of nonlinear optical materials over the past century. From the atomic scale, the electron configuration of the boron atom is 1*s*^2^2*s*^2^2*p*^1^, indicating the presence of one *p* orbital electron and two unoccupied *p* orbitals. Therefore, the boron atom can function as an electron acceptor to interact with oxygen atoms, resulting in the formation of linear [BO_2_], triangular [BO_3_], and tetrahedral [BO_4_] basic units. And these basic units can be further polymerized to generate a diverse array of borate structures. With the continuous efforts of scientists worldwide, numerous borates have been discovered in recent years. For example, if the founded borates are arranged in ascending order according to the number of boron atoms in the formula, there are NaBO_2_,^6^ BaB_2_O_4_, LiB_3_O_5_, AB_4_O_6_F (A = NH_4_, Na, Rb, Cs),^7−10^ MB_5_O_7_F_3_(M = Mg, Ca, Sr),^11−13^ A_2_B_6_O_9_F_2_ (A = Li, Na, K, NH_4_),^14−19^ Rb_3_B_7_O_12_,^20^ BaB_8_O_13_,^21^ CsB_9_O_14_,^22^ Ba_2_B_10_O_17_,^23^ NH_4_B_11_O_16_(OH)_2_,^24^ (NH_4_)_4_[B_12_O_16_F_4_(OH)_4_],^25^ K_10_B_13_O_15_F_19_,^26^ Sr_3_B_14_O_24_,^27^ NH_4_B_15_O_20_(OH)_8_·4H_2_O,^28^ Sr_2_B_16_O_26_,^29^ Sr_8_MgB_18_O_36_,^30^ Rb_5_B_19_O_31_,^31^ Na_2_Cs_2_B_20_O_32_,^32^ NaCsB_21_O_36_,^33^ etc. Among them, compounds containing the 12-membered ring [B_12_O_24_] unit are popular. There are six [BO_4_] and six [BO_3_] on the inside and outside of the [B_12_O_24_] unit, respectively. And it can also be viewed as six [B_3_O_8_] units up-and-down crossly connected by sharing [BO_4_] unit. According to the theory of Christ et al.^34^ and Burns et al.,^35^ the descriptor of the 12-membered ring [B_12_O_24_] unit can be expressed as 12:6Δ + 6T and 6Δ6□:≪Δ2□>-•>. As far as current knowledge goes, Na_8_[B_12_O_20_(OH)_4_] was the first borate to contain the [B_12_O_24_] unit, discovered in 1979.^36^ Subsequently, silver borate Ag_6_[B_12_O_18_(OH)_6_]·3H_2_O was found in 1990,^37^ followed by zinc borate Zn(H_2_O)B_2_O_4_·*x*H_2_O^38^ in 2002 and K_7_{(BO_3_)Mn[B_12_O_18_(OH)_6_]}·H_2_O in 2004.^39^ And Li_3_NaBaB_6_O_12_,^41^ Li_3_KB_4_O_8_, LiNa_2_Sr_8_B_12_O_24_F_6_Cl,^42^ Li_7_Na_2_KRb_2_B_12_O_24_, Li_7.35_Na_2.36_K_1.50_Cs_0.78_B_12_O_24_, Li_6.97_Na_2.63_K_1.24_Cs_1.15_B_12_O_24_, Li_7.27_Na_2.67_Rb_2.06_B_12_O_24_,^43^ Ca_3_Na_4_LiBe_4_B_10_O_24_F,^44^ Sr_3_LiNa_4_Be_4_B_10_O_24_F,^45^ and Li_6.58_Na_7.43_Sr_4_(B_9_O_18_)(B_12_O_24_)Cl^46^ were all discovered after 2010. The majority of the aforementioned compounds are anhydrous borates featuring the [B_12_O_24_] unit. Additionally, most of these structures exhibit centrosymmetry and are arranged in a nonparallel, isolated manner.

In this paper, three polyborates featuring [B_12_O_24_] unit were synthesized via the high-temperature solution method. Their anionic groups exhibit distinct dimensions. LiNa_11_B_28_O_48_ features a novel 3[B_28_O_48_]_∞_ framework anion structure, which is constructed by [B_12_O_24_], [B_15_O_30_], and [BO_3_] units through sharing of corner O atoms. Li_1.45_Na_7.55_B_21_O_36_ exhibits a unique 1[B_21_O_36_]_∞_ chain anion structure, built by [B_12_O_24_] and [B_9_O_18_] units through sharing of corner O atoms. New FBBs, namely, [B_15_O_30_] and [B_21_O_39_], have been identified in LiNa_11_B_28_O_48_ and Li_1.45_Na_7.55_B_21_O_36_, respectively. Li_2_Na_4_Ca_7_Sr_2_B_13_O_27_F_9_ is a borate with an isolated anion structure composed of [B_12_O_24_] and [BO_3_] units. Furthermore, we conducted a detailed comparison of these structures with other borates containing the [B_12_O_24_] unit in order to investigate their structural commonalities and differences. The thermal stability and optical properties of these three compounds were evaluated to assess their suitability for further application.

## Experimental Section

### Single-Crystal Synthesis

Single crystals of LiNa_11_B_28_O_48_, Li_1.45_Na_7.55_B_21_O_36_, and Li_2_Na_4_Ca_7_Sr_2_B_13_O_27_F_9_ were obtained via the high-temperature solution method with specific synthesis details as follows:

#### LiNa_11_B_28_O_48_

A mixture of LiBO_2_ (0.079 g, 0.99 mmol), NaF (0.161 g, 3.83 mmol), and H_3_BO_3_ (0.125 g, 2.02 mmol) was enclosed in a clean silica quartz tube with dimensions of Φ10 mm × 100 mm. The NaF served as both a co-solvent and the source of Na in LiNa_11_B_28_O_48_. The sample was gradually heated to 650 °C over a period of 24 h and held at this temperature for an additional 48 h to ensure complete melting. Subsequently, the solution was slowly cooled to room temperature at a rate of 1.5 °C/h. Finally, the colorless crystals of LiNa_11_B_28_O_48_ can be selectively harvested from the resulting product.

#### Li_1.45_Na_7.55_B_21_O_36_

A mixture of LiBF_4_ (0.316 g, 3.37 mmol), NaF (0.47 g, 11.19 mmol), Y_2_O_3_ (0.291 g, 1.29 mmol), and B_2_O_3_ (0.975 g, 14.00 mmol) was charged into a platinum crucible wherein Y_2_O_3_ acted as the catalyst for the reaction while NaF served as both co-solvent and source of Na for Li_1.45_Na_7.55_B_21_O_36_. The samples were gradually heated at a rate of 100 °C/h from room temperature to 750 °C and held at this temperature for 24 h to guarantee full dissolution. Subsequently, the solution was slowly cooled down to 500 °C with a cooling rate of 1.5 °C/h, followed by rapid cooling to room temperature at a rate of 5 °C/h. Finally, small transparent thin crystals of Li_1.45_Na_7.55_B_21_O_36_ were picked out from the obtained samples.

#### Li_2_Na_4_Ca_7_Sr_2_B_13_O_27_F_9_

Li_2_Na_4_Ca_7_Sr_2_B_13_O_27_F_9_ was synthesized using a similar process as Li_1.45_Na_7.55_B_21_O_36_, with a mixture of LiF (0.108 g, 4.16 mmol), Na_2_CO_3_ (0.184 g, 1.74 mmol), CaF_2_ (0.267 g, 3.42 mmol), SrF_2_ (0.443 g, 3.53 mmol), H_3_BO_3_ (0.553 g, 8.94 mmol), PbO (0.194 g, 0.87 mmol), and PbF_2_ (0.292 g, 1.19 mmol). The inclusion of PbO and PbF_2_ in the reaction served as cosolvents but were not incorporated into the final structure.

### Polycrystalline Powder Synthesis

Polycrystalline samples of these three compounds can be synthesized via the high-temperature solid-state reactions as the equation below
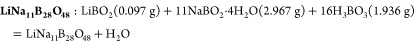

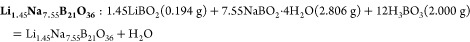

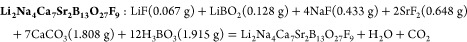


The initial raw materials, in stoichiometric ratio as per the reaction equation of three compounds mentioned above, were thoroughly mixed and filled into a ceramic crucible. Then, the samples were gradually heated to 300 °C over a period of 6 h and maintained at that temperature for 10 h in order to decompose H_3_BO_3_ and remove gases such as H_2_O and CO_2_. Subsequently, the preheated mixture was subjected to heated to 640 °C and held at that temperature for 72 h with intermittent grinding and mixing. Finally, turn off the stove and allow the sample to cool naturally. Pure polycrystalline powder of LiNa_11_B_28_O_48_ and Li_1.45_Na_7.55_B_21_O_36_ were obtained. However, as shown in Figure 5, despite our best efforts, the polycrystalline powder of Li_2_Na_4_Ca_7_Sr_2_B_13_O_27_F_9_ we obtained was not entirely pure.

### Powder X-ray Diffraction

The purity of the polycrystalline powders of these three compounds was assessed using a Bruker D2 PHASER powder X-ray diffractometer (PXRD). Diffraction data were collected over a 2θ range of 5–70°, with a scan rate of 0.05 s/step and a scan step size of 0.02°.

### Single-Crystal X-ray Diffraction

High-quality colorless single crystals were selected for single-crystal X-ray diffraction (XRD) tests. The diffraction data were collected using a Bruker D8 VENTURE single-crystal X-ray diffractometer with Cu Kα radiation (λ = 1.54178 Å) at 297.15 K for LiNa_11_B_28_O_48_ and Mo Kα radiation (λ = 0.71073 Å) at 273.15 K for Li_1.45_Na_7.55_B_21_O_36_, Li_2_Na_4_Ca_7_Sr_2_B_13_O_27_F_9_. The APEX3 software, which is compatible with the diffractometer, was utilized for data reduction and analysis. These structures were solved with the SHELXT program through the intrinsic phasing method, refined by the full matrix least squares method in the SHELXL program, and checked for symmetry via PLATON program in Olex2. Table 1 provides detailed information on crystal and structure refinement data. The final atomic coordinates, equivalent isotropic displacement parameters, bond valence sum (BVS) for each atom, as well as the selected bond lengths and angles in compounds are listed in Tables S1–S9 in the Supplementary Information (ESI).

**Table 1:** Crystal and Structure Refinement Data

empirical formula	LiNa_11_B_28_O_48_	Li_1.45_Na_7.55_B_21_O_36_	Li_2_Na_4_Ca_7_Sr_2_B_13_O_27_F_9_
temperature	297.15 K	273.15 K	273.15 K
crystal system, space group	hexagonal, *P*6_3_/*mcm*	hexagonal, *P*6_3_/*mcm*	hexagonal, *P*6_3_/*m*
unit cell dimensions	*a* = 9.50430(10) Å	*a* = 9.3446(3) Å	*a* = 9.32940(10) Å
	*b* = 9.50430(10) Å	*b* = 9.3446(3) Å	*b* = 9.32940(10) Å
	*c* = 25.0041(3) Å	*c* = 20.2013(10) Å	*b* = 19.5978(5) Å
volume	1956.06(5) Å^3^	1527.68(12) Å^3^	1477.22(5) Å^3^
*Z*, calculated density	2, 2.259 g·cm^–3^	2, 2.145 g·cm^–3^	2, 2.934 g·cm^–3^
crystal size	0.059 × 0.078 × 0.081 mm^3^	0.11 × 0.12 × 0.15 mm^3^	0.102 × 0.103 × 0.112 mm^3^
absorption coefficient	2.894 mm^–1^	0.286 mm^–1^	5.053 mm^–1^
*F* (000)	1296	961	1256
radiation	Cu Kα (λ = 1.54178)	Mo Kα (λ = 0.71073)	Mo Kα (λ = 0.71073)
θ range for data collection	3.54 to 68.3°	2.016 to 17.483°	2.016 to 17.483°
limiting indices	–11 ≤ *h* ≤ 10, –9 ≤ *k* ≤ 11, –19 ≤ *l* ≤ 30	–12 ≤ *h* ≤ 12, –11 ≤ *k* ≤ 12, –26 ≤ *l* ≤ 26	–9 ≤ *h* ≤ 12, –12 ≤ *k* ≤ 12, –25 ≤ *l* ≤ 25
reflections collected/unique	14 995/688 [*R*_int_ = 0.0578]	11 789/669 [*R*_int_ = 0.0862]	9997/1164 [*R*_int_ = 0.0390]
data/restraints/parameters	688/6/96	669/0/69	1164/0/104
goodness-of-fit on *F*^2^	1.216	1.031	1.155
final *R* indices [*F*_o_^2^ > 2σ(*F*_o_^2^)]^a^	*R*_1_ = 0.0625, w*R*_2_ = 0.1347	*R*_1_ = 0.0321, w**R**_2_ = 0.0851	*R*_1_ = 0.0290, w*R*_2_ = 0.0769
largest diff. peak and hole	0.44 and –0.63 e·Å^–3^	0.91 and –0.76 e·Å^–3^	1.09 and –0.68 e·Å^–3^

a *R*_1_ = ∑||*F*_o_| – |*F*|| / ∑|*F*_o_| and *w*R**_2_ = [∑*w*(*F*_o_^2^ – *F*_c_^2^)^2^ / ∑*wF*_o_^4^]^1/2^ for *F*_o_^2^ > 2σ(*F*_o_^2^).

### Infrared Spectroscopy

A Shimadzu IR Affinity-11 FT-IR spectrometer was utilized for the infrared (IR) spectroscopy analysis to identify characteristic peaks and verify the rationality of these three structures. Data were collected within a range of 400 to 4000 cm^–1^ with a resolution set at 1 cm^–1^. These samples were homogeneously mixed with dried KBr at a ratio of 1:100 for testing purposes, while pure KBr was employed to eliminate air noise-induced peaks.

### Energy-Dispersive X-ray Spectroscopy

Elemental analysis was used to identify the constituent elements of the crystal composition. The test was performed utilizing a BRUKER X-flash-sdd-5010 energy-dispersive X-ray spectroscope (EDS) integrated into a SUPRA 55 VP field emission scanning electron microscope (SEM).

### UV–Vis–NIR Diffuse Reflectance Spectroscopy

The transmittance range of these compounds was evaluated using UV–vis–NIR diffuse reflectance spectroscopy, with a Shimadzu SolidSpec-3700DUV spectrophotometer employed for the test. Data were collected at room temperature over a wavelength range of 200–2600 nm, with tetrafluoroethylene serving as the standard sample for the diffuse reflectance test.

### TG-DSC Analyses

Thermogravimetry (TG) and differential scanning calorimetry (DSC) tests were performed to investigate the thermal stability of these three compounds. The simultaneous NETZSCH STA 449 F3 thermal analyzer instrument was utilized for testing, with samples of the three compounds placed in a Pt crucible and heated from 40 to 800 °C at a rate of 5 °C·min^–1^ under a nitrogen atmosphere. The equipment recorded weight and thermal energy changes as the temperature changed.

## Results and Discussion

### Structural Structure

**Figure 1:**
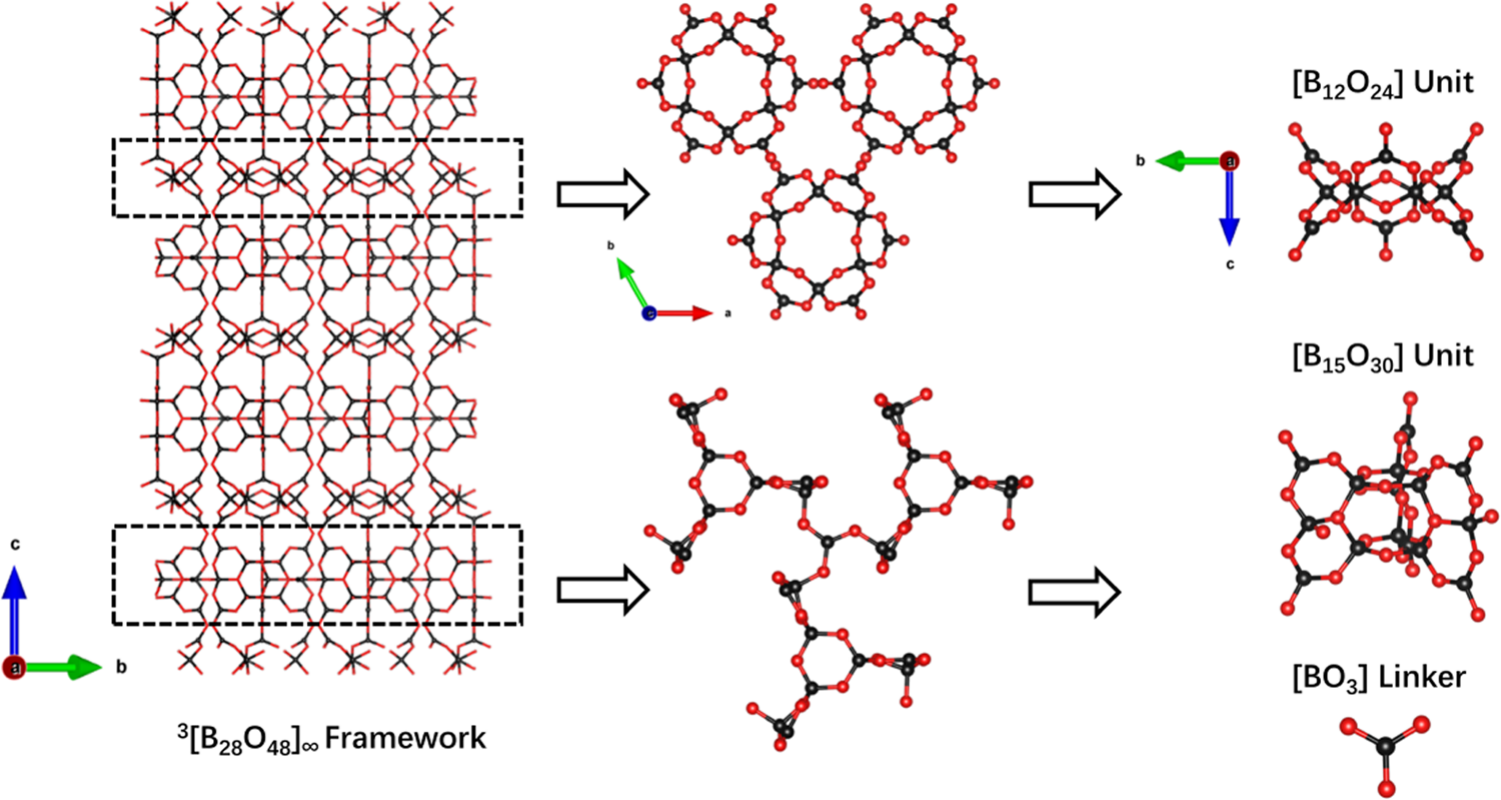
Anion group in three dimensions and the novel FBB [B_15_O_30_] in LiNa_11_B_28_O_48_.

LiNa_11_B_28_O_48_ crystallizes in a hexagonal crystal system with space group *P*6_3_/*mcm* (No. 193). LiNa_11_B_28_O_48_ is composed of [LiO_6_], [NaO_6_], [NaO_7_], [BO_3_], and [BO_4_] units. [BO_3_] and [BO_4_] connect through corner O atoms to form polyborates of [B_15_O_30_] and [B_12_O_24_]. To the best of our knowledge, the [B_15_O_30_] unit represents a novel fundamental building block (FBB). Moreover, through sharing corner O atoms, [B_15_O_30_] and [B_12_O_24_] construct tunnels of [B_27_O_51_]. These tunnels are further linked by [BO_3_] to form the overall anionic framework of 3[B_28_O_48_]_∞_ in LiNa_11_B_28_O_48_ (Figure 1). In the structure, the B–O bond length in [BO_3_] units ranges from 1.35 to 1.49 Å, whereas the B–O bond length in [BO_4_] units ranges from 1.43 to 1.59 Å. The Li atoms are coordinated by six oxygen atoms to form [LiO_6_] octahedra, with a constant Li–O bond distance of 2.11 Å. The Na atoms exhibit two distinct coordination environments, namely, [NaO_6_] and [NaO_7_] polyhedra, with Na–O bond distances ranging from 2.23 to 2.93 Å (Figure S1).

**Figure 2:**
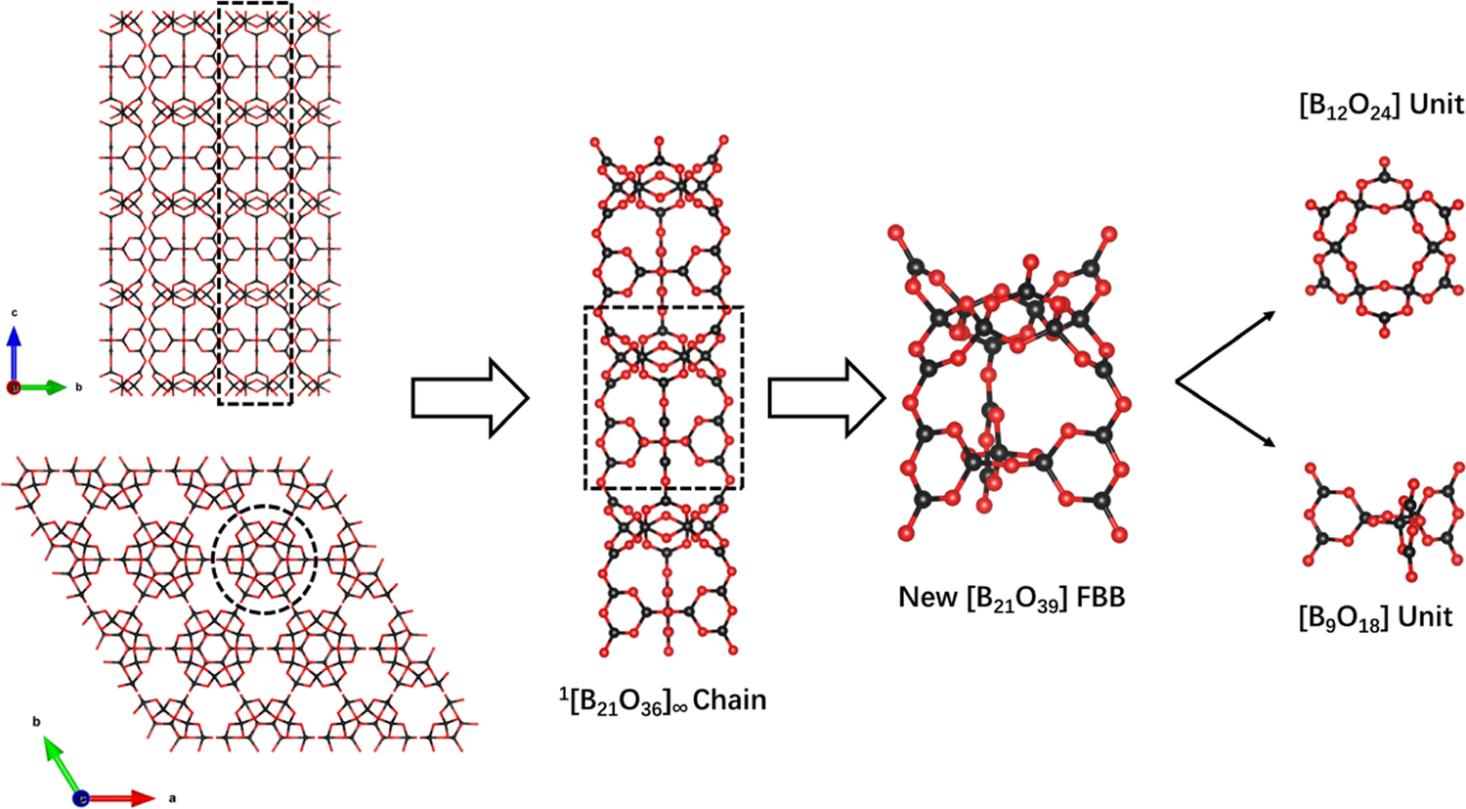
Anion group in one dimension and the novel FBB [B_21_O_39_] in Li_1.45_Na_7.55_B_21_O_36_.

Li_1.45_Na_7.55_B_21_O_36_ crystallizes in the hexagonal crystal system with space group *P*6_3_/*mcm* (No. 193). Li_1.45_Na_7.55_B_21_O_36_ is composed of [LiO_6_], [Na(Li)O_6_], [NaO_7_], [BO_3_], and [BO_4_] units. [BO_3_] and [BO_4_] connect to form polyborates of [B_9_O_18_] and [B_12_O_24_] by sharing corner O atoms. Furthermore, these polyborates combine to create an FBB of [B_21_O_39_]. It should be noted that this particular FBB has not been previously reported. These FBBs are interconnected to form the anionic chain of 1[B_21_O_36_]_∞_ in Li_1.45_Na_7.55_B_21_O_36_ (Figure 2). Interestingly, it differs from that in the recently published compound Li_6.58_Na_7.43_Sr_4_(B_9_O_18_)(B_12_O_24_)Cl, where [B_9_O_18_] and [B_12_O_24_] units exist as isolated clusters. In the structure, [BO_3_] units with three-fold coordination exhibit B–O bond distances ranging from 1.34 to 1.42 Å, while [BO_4_] units with four-fold coordination possess B–O bond distances within the range of 1.44–1.51 Å. The bond distances of Na(Li)–O in [Na(Li)O_6_] octahedra exhibit a range from 2.300 to 2.301 Å, while the Li–O bond lengths in [LiO_6_] octahedra remain constant at 2.101 Å. The Na–O bond distances in [NaO_7_] vary from 2.291 to 2.917 Å (Figure S2).

**Figure 3:**
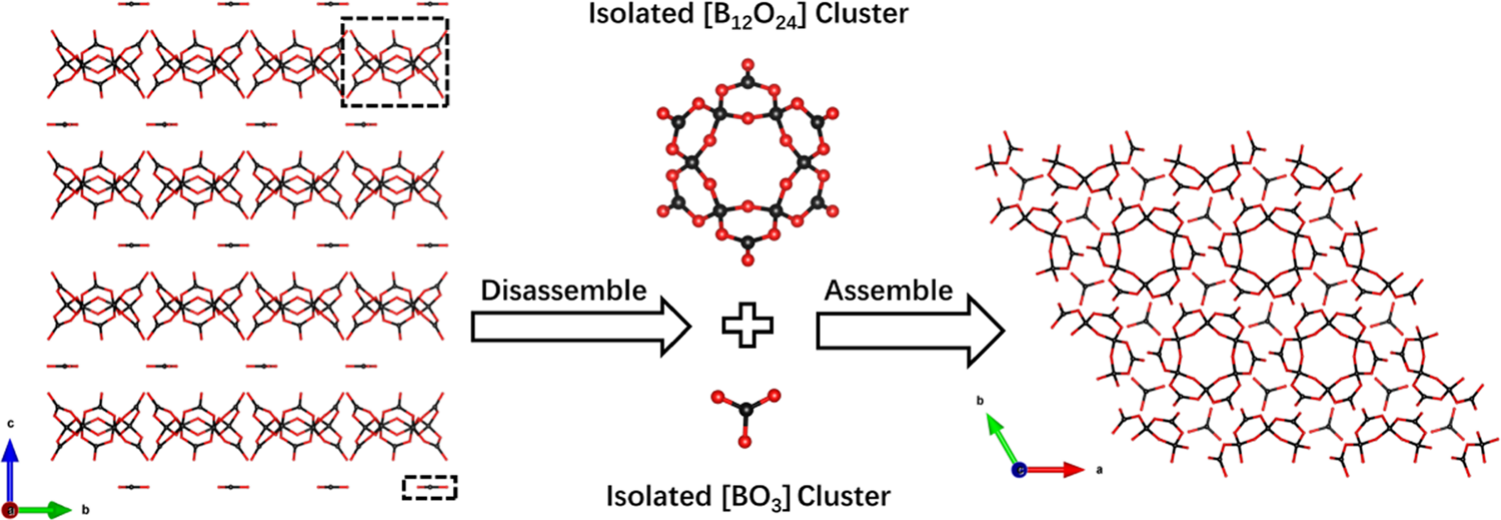
Anion group in zero dimension in Li_2_Na_4_Ca_7_Sr_2_B_13_O_27_F_9_.

Li_2_Na_4_Ca_7_Sr_2_B_13_O_27_F_9_ crystallizes in the hexagonal crystal system with space group *P*6_3_/*m* (No. 176). Li_2_Na_4_Ca_7_Sr_2_B_13_O_27_F_9_ is composed of [LiO_6_], [LiF_6_], [NaO_6_], [CaO_4_F_4_], [Ca(Sr)O_5_F_3_], [BO_3_], and [B_12_O_24_] units. In contrast to the preceding two compounds, Li_2_Na_4_Ca_7_Sr_2_B_13_O_27_F_9_ features discrete anionic groups in the form of [BO_3_] and [B_12_O_24_] units (Figure 3). In the structure, the B–O bond distances within the [BO_3_] units exhibit a range of 1.33–1.41 Å, while those within the [BO_4_] units display a range of 1.46 to 1.49 Å (Figure S3). The Li–O bond and Li–F bond distances in [LiO_6_] and [LiF_6_] octahedra take the constant value of 2.104 and 2.103 Å, respectively. The bond distances of Na–O in [NaO_6_] exhibit a range of 2.299 to 2.588 Å. In [Ca(Sr)O_5_F_3_], the bond distances of Ca(Sr)–O stretch from 2.42 to 3.24 Å, while the Ca(Sr)–F vary from 2.34 to 2.56 Å. Similarly, the Ca–O bond distances vary from 2.36 to 2.70 Å and Ca–F bond stretch from 2.39 to 2.48 Å in [CaO_4_F_4_] (Figure S4).

These three compounds all contain high-symmetry 12-membered ring [B_12_O_24_] units. Through an investigation of published borates containing [B_12_O_24_] units, a total of 20 cases have been identified to date (including the three compounds in this study) (Table 2). As depicted in the table, fundamental crystal structure data have been succinctly summarized, and for ease of subsequent discourse, Arabic numerals were assigned to the 20 compounds. Notably, intriguing patterns exist among these borates featuring the ring-like [B_12_O_24_] unit. First, with the exception of hydroxyl-containing borates (No.1–5) and recently discovered borates (No. 17–20), most crystallize in the same *R*3̅ space group (No.6–16). Previous research and analysis suggest that this is primarily due to the local symmetry 3̅ of the [B_12_O_24_] units within their structure.^46^ And for exceptional cases, it is primarily due to the introduction of hydroxyl or other group that can disrupt the symmetry of [B_12_O_24_] units, such as the [B_12_O_20_(OH)_4_] unit in Na_8_[B_12_O_20_(OH)_4_], the [B_12_O_18_(OH)_6_] unit in Ag_6_[B_12_O_18_(OH)_6_]·3H_2_O. In this study, LiNa_11_B_28_O_48_, Li_1.45_Na_7.55_B_21_O_36_ have a space group of *P*6_3_/*mcm* and the space group of Li_2_Na_4_Ca_7_Sr_2_B_13_O_27_F_9_ is *P*6_3_/*m*. Likewise, it is [B_15_O_30_] unit in LiNa_11_B_28_O_48_, [B_9_O_18_] unit in Li_1.45_Na_7.55_B_21_O_36_, and [BO_3_] unit in Li_2_Na_4_Ca_7_Sr_2_B_13_O_27_F_9_ that break the symmetry of [B_12_O_24_] units and change the space group of compounds. Second, except compounds of No. 1–5, the values of *a* and *b* are equivalent, both exceeding 9 Å, and the value of *c* is approximately 20 Å, which significantly surpasses *a* and *b* in their cell parameters. This pertains to the crystal system of the crystallographic space group of these compounds. Third, this series of compounds with the ring-like [B_12_O_24_] units all contain Li and Na elements with the exception of compounds No. 2–5. Moreover, the anion groups of these compounds exhibit distinct dimensions and most of them are isolated structures. Based on the borate anion dimension theory proposed by P. Becker and N. I. Leonyuk,^48,49^ the dimension of borate anion groups is often influenced by the M/B ratio, with a higher M/B ratio leading to a smaller dimension of the anion group (where M and the B represent the stoichiometric number of metal and boron in this compound). The borates containing [B_12_O_24_] units also adhere to this principle. As indicated in the penultimate column of Table 2, with the exception of LiNa_11_B_28_O_48_ and Li_1.45_Na_7.55_B_21_O_36_ discovered in this study, all other borates listed have isolated anion groups, and their M/B ratios are greater than 1/2. The LiNa_11_B_28_O_48_ and Li_1.45_Na_7.55_B_21_O_36_ exhibit a three-dimensional framework structure and a one-dimensional chain structure, with both having an M/B ratio of 3/7, which is lower than that of the isolated ones.

**Table 2:** Basic Information of Inorganic Borates with Ring-like B_12_O_24_ Clusters

no.	chemical formula	space group	B–O units	cell parameters	M/B	refs
1	Na_8_[B_12_O_20_(OH)_4_]	**P*21/*c**(14)	[B_12_O_20_(OH)_4_]	*a* = 8.709 Å; *b* = 11.917 Å; *c* = 9.468 Å	2/3	(36)
				α = γ = 90°; β = 96.02°		
2	Ag_6_[B_12_O_18_(OH)_6_]·3H_2_O	**P*21*/**c**(14)	[B_12_O_18_(OH)_6_]	*a* = 11.784 Å; *b* = 10.654 Å; *c* = 9.468 Å	1/2	(37)
				α = γ = 90°; β = 96.02°		
3	Zn(H_2_O)B_2_O_4_·*x*H_2_O(*x* ≈ 0.12)	*R*3̅*m*(166)	[B_12_O_24_]	*a* = *b* = 11.410 Å; *c* = 17.156Å	1/2	(38)
				α = β = 90°; γ = 120°		
4	K_7_{(BO_3_)Mn[B_12_O_18_(OH)_6_]}·H_2_O	*Pmn*2_1_(31)	[B_12_O_20_(OH)_4_] + [BO_3_]	*a* = 12.397 Å; *b* = 9.1185 Å; *c* = 13.068 Å	8/13	(39)
				α = β = γ = 90°		
5	K_7_{(BO_3_)Zn[B_12_O_18_(OH)_6_]}·H_2_O	*Pmn*2_1_(31)	[B_12_O_20_(OH)_4_] + [BO_3_]	*a* = 12.365 Å; *b* = 9.096 Å; *c* = 13.041 Å	8/13	(40)
				α = β = γ = 90°		
6	Li_3_NaBaB_6_O_12_	*R*3̅(148)	[B_12_O_24_]	*a* = *b* = 9.462 Å; *c* = 18.71 Å	5/6	(41)
				α = β = 90°; γ = 120°		
7	Li_3_KB_4_O_8_	*R*3̅(148)	[B_12_O_24_]	*a* = *b* = 9.211 Å; *c* = 19.705 Å	1/1	(42)
				α = β = 90°; γ = 120°		
8	LiNa_2_Sr_8_B_12_O_24_F_6_Cl	*R*3̅(148)	[B_12_O_24_]	*a* = *b* = 9.677 Å; *c* = 24.30 Å	11/12	(42)
				α = β = 90°; γ = 120°		
9	Ca_3_Na_4_LiBe_4_B_10_O_24_F	*R*3̅(148)	[B_12_O_24_] + [BO_3_]	*a* = *b* = 9.354 Å; *c* = 38.053 Å	6/5	(44)
				α = β = 90°; γ = 120°		
10	Sr_3_LiNa_4_Be_4_B_10_O_24_F	*R*3̅(148)	[B_12_O_24_] + [BO_3_]	*a* = *b* = 9.4645 Å; *c* = 38.842 Å	6/5	(45)
				α = β = 90°; γ = 120°		
11	Cd_3_LiNa_4_Be_4_B_10_O_24_F	*R*3̅(148)	[B_12_O_24_] + [BO_3_]	*a* = *b* = 9.302 Å; *c* = 37.782 Å	6/5	(45)
				α = β = 90°; γ = 120°		
12	Na_5_Li[B_12_O_18_(OH)_6_]·2H_2_O	*R*3̅*c*(167)	[B_12_O_18_(OH)_6_]	*a* = *b* = 9.677 Å; *c* = 36.358 Å	1/2	(47)
				α = β = 90°; γ = 120°		
13	Li_7_Na_2_KRb_2_B_12_O_24_	*R*3̅(148)	[B_12_O_24_]	*a* = *b* = 9.548 Å; *c* = 19.55 Å	1	(43)
				α = β = 90°; γ = 120°		
14	Li_7.35_Na_2.36_K_1.50_Cs_0.78_B_12_O_24_	*R*3̅(148)	[B_12_O_24_]	*a* = *b* = 9.479 Å; *c* = 19.493 Å	1	(43)
				α = β = 90°; γ = 120°		
15	Li_6.97_Na_2.63_K_1.24_Cs_1.15_B_12_O_24_	*R*3̅(148)	[B_12_O_24_]	*a* = *b* = 9.530 Å; *c* = 19.534 Å	1	(43)
				α = β = 90°; γ = 120°		
16	Li_7.27_Na_2.67_Rb_2.06_B_12_O_24_	*R*3̅(148)	[B_12_O_24_]	*a* = *b* = 9.453 Å; *c* = 19.413 Å	1	(43)
				α = β = 90°; γ = 120°		
17	Li_6.58_Na_7.43_Sr_4_(B_9_O_18_)(B_12_O_24_)Cl	*P*6_3_/*m*(176)	[B_12_O_24_] + [B_9_O_18_]	*a* = *b* = 9.305 Å; *c* = 24.324 Å	6/7	(46)
				α = β = 90°; γ = 120°		
18	LiNa_11_B_28_O_48_	*P*6_3_/*mcm*(193)	[B_12_O_24_ + B_15_O_30_ + BO_3_]	*a* = *b* = 9.504 Å; *c* = 25.0038 Å	3/7	this work
				α = β = 90°; γ = 120°		
19	Li_1.45_Na_7.55_B_21_O_36_	*P*6_3_/*mcm*(193)	[B_12_O_24_ + B_9_O_18_]	*a* = *b* = 9.345 Å; *c* = 20.201 Å	3/7	this work
				α = β = 90°; γ = 120°		
20	Li_2_Na_4_Ca_7_Sr_2_B_13_O_27_F_9_	*P*6_3_/*m*(176)	[B_12_O_24_] + [BO_3_]	*a* = *b* = 9.329 Å; *c* = 19.598 Å	15/13	this work
				α = β = 90°; γ = 120°		

**Figure 4:**
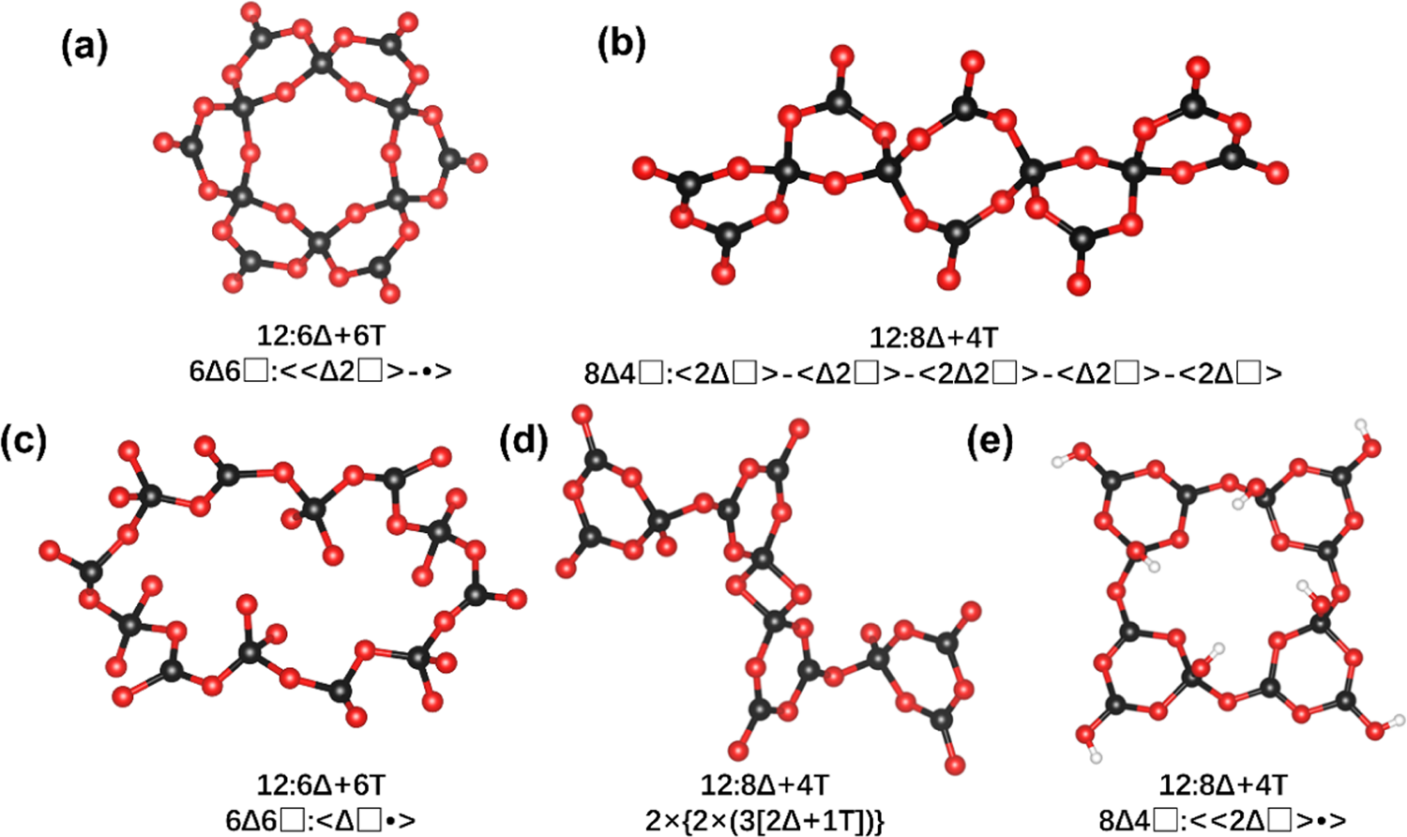
Different [B_12_O_24_] units and their topological representation in (a) Li_3_KB_4_O_8_, (b) Cs_3_AlB_6_O_12_, (c) Ba_4_Na_2_Zn_4_(B_3_O_6_)_2_(B_12_O_24_), (d) Ca_9_B_26_O_34_(OH)_24_Cl_4_·13H_2_O, and (e) KB_3_O_4_(OH)_2_.

During the survey process, four additional configurations of [B_12_O_24_] units were also discovered in Cs_3_AlB_6_O_12_,^50^ Ba_4_Na_2_Zn_4_(B_3_O_6_)_2_(B_12_O_24_),^51^ pringleite mineral Ca_9_B_26_O_34_(OH)_24_Cl_4_·13H_2_O,^52^ and KB_3_O_4_(OH)_2_.^53^ They differ from the [B_12_O_24_] units mentioned in this paper. As depicted in Figure 4, these five distinct [B_12_O_24_] units exhibit varying ring-shaped structures composed of twelve boron atoms and twenty-four oxygen atoms. However, the connectivity patterns among these constituent elements differ significantly. The [B_12_O_24_] unit in Ba_4_Na_2_Zn_4_(B_3_O_6_)_2_(B_12_O_24_) (Figure 4c) and this paper (Figure 4a) are both made up of six [BO_3_] and six [BO_4_], so the topological descriptor of them can be represented as 12:8Δ + 4T using the method of Christ and Clark. However, the [B_12_O_24_] unit in this study is composed of one 12-membered ring and six 6-membered rings and can be represented as 6Δ6□:≪Δ2□>-•> on the description theory of Burns. The [B_12_O_24_] unit of Ba_4_Na_2_Zn_4_(B_3_O_6_)_2_(B_12_O_24_) (Figure 4c) expands to a 24-membered ring and can be described as 6Δ6□:<Δ□•>_._ The [B_12_O_24_] units in Cs_3_AlB_6_O_12_ (Figure 4b), Ca_9_B_26_O_34_(OH)_24_Cl_4_·13H_2_O (Figure 4d), and KB_3_O_4_(OH)_2_ (Figure 4e) are constructed by eight [BO_3_] and four [BO_4_]; thus, they can be expressed as 12:8Δ + 4T according to the method of Christ and Clark. However, they exhibit significant differences with several distinct rings. The [B_12_O_24_] unit in Cs_3_AlB_6_O_12_ contains four 6-membered rings and one 8-membered ring; thus, the detailed descriptor of the structure is 8Δ4□:<2Δ□>-<Δ2□>-<2Δ2□>-<Δ2□>-<2Δ□> (Figure 4b). The [B_12_O_24_] unit in Ca_9_B_26_O_34_(OH)_24_Cl_4_·13H_2_O is composed of four 6-membered rings and one 4-membered ring, which can be represented as 2×{2×(3[2Δ + 1T])} (Figure 4d). And the [B_12_O_24_] unit in KB_3_O_4_(OH)_2_ is constructed by four 6-membered rings and one 16-membered ring; thus, the detailed descriptor is 8Δ4□:≪2Δ□>•>. Additionally, hydrogen atoms are bonded to the dangling oxygen atoms within this unit (Figure 4e). To have a picture of all these [B_12_O_24_] units exhibiting distinct structures, the [B_12_O_24_] unit present in our three compounds demonstrates the highest degree of symmetry (Figure 4a).

### Phase Identification and Analysis

**Figure 5:**
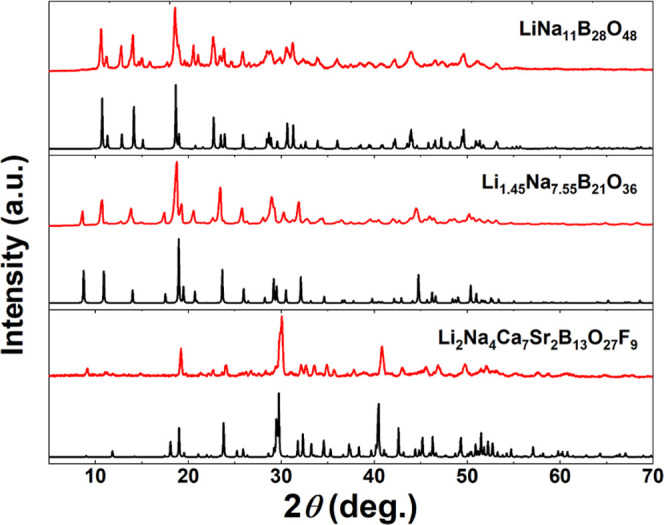
Calculated (black lines) and experimental (red lines) powder XRD patterns of LiNa_11_B_28_O_48_, Li_1.45_Na_7.55_B_21_O_36_, and Li_2_Na_4_Ca_7_Sr_2_B_13_O_27_F_9_.

The bond valence sum (Tables S1, S4, and S7) and conventional structural configuration of three- and four-coordinated B–O units (Tables S2, S5, and S8) provide preliminary confirmation of the structural rationality of the three compounds. Powder XRD confirmed the phase purity of these samples (Figure 5). And as shown in Figure S5, the IR spectrum well verified the existence of [BO_3_] and [BO_4_] units in structures, which was consistent with results obtained from single-crystal XRD analysis. According to previous research,^54^ the stretching vibration signals of the [BO_3_] can be observed within the spectral range of 1396–1419 cm^–1^. The asymmetric stretching signals of the B–O bond in [BO_3_] appear between 1234 and 1273 cm^–1^. The asymmetric stretching signals of the B–O bond in [BO_4_] can be observed within the range of 1026–1041 cm^–1^, while the symmetric stretching signals of the B–O bond in [BO_3_] are present between 887 and 964 cm^–1^. Additionally, out-of-plane bending modes of both [BO_3_] and [BO_4_] can be detected within a range of 628 to 740 cm^–1^. The elemental analysis revealed the presence of fluorine elements in Li_2_Na_4_Ca_7_Sr_2_B_13_O_27_F_9_ (Figure S6).

### Thermal and Optical Properties

**Figure 6:**
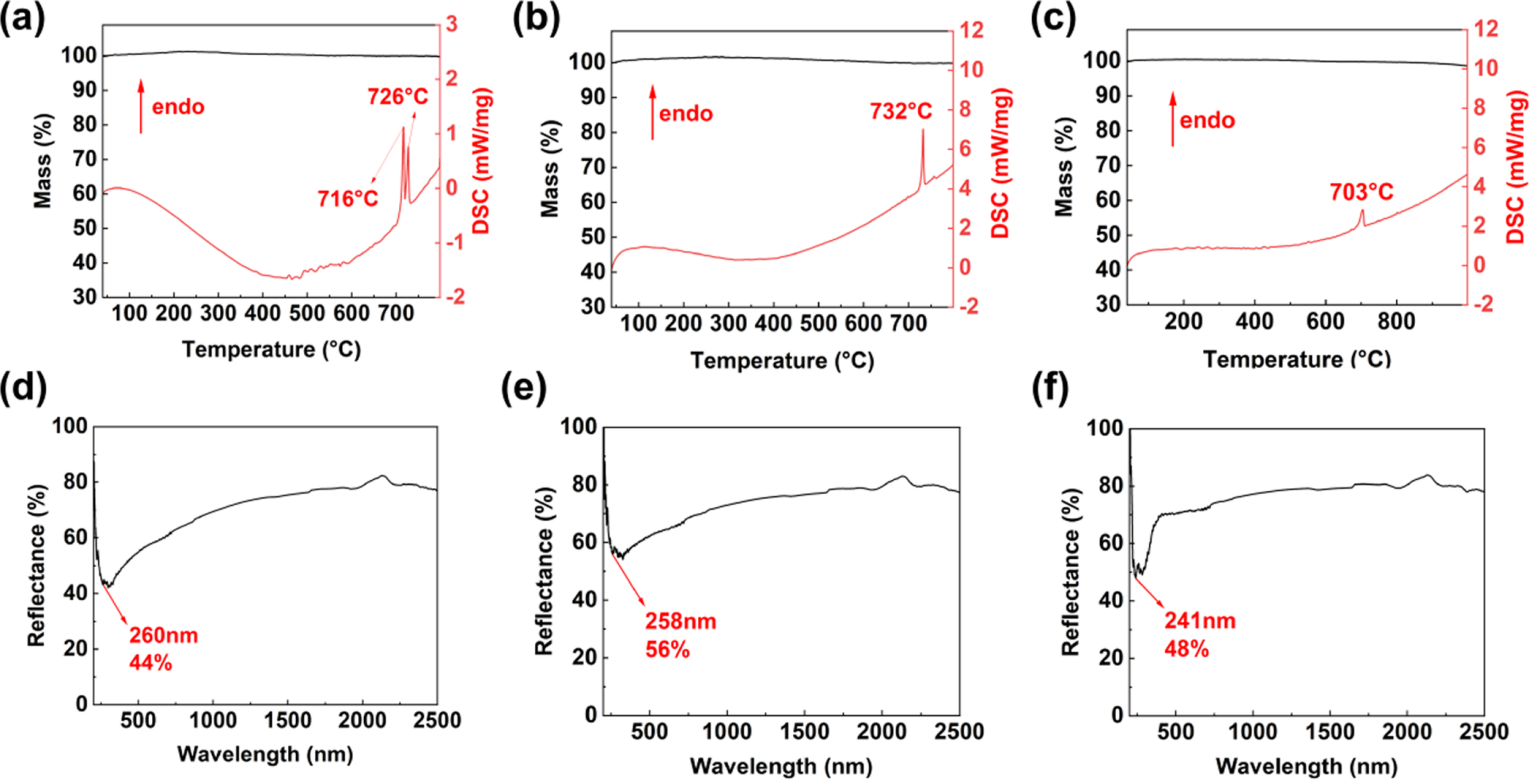
TG-DSC curves and UV–vis–NIR diffuse reflectance spectra of LiNa_11_B_28_O_48_ (a, d), Li_1.45_Na_7.55_B_21_O_36_ (b, e), and Li_2_Na_4_Ca_7_Sr_2_B_13_O_27_F_9_ (c, f).

The thermostability test results of samples are depicted in the TG and DSC curves (Figure 6). The TG curves of the three samples exhibited no significant decrease in their curves, indicating that there was no substantial weight loss during the heating process. Additionally, distinct endothermic peaks were observed for all three compounds after 700 °C in the DSC curves, demonstrating their thermal stability up to this temperature. The diffuse reflectance spectra of the three compounds exhibit negligible absorption within the range of 266–2500 nm, indicating their high ultraviolet transmittance among these regions (Figure 6). There are two explanations for this phenomenon: (1) The absence of *d–d* and *f–f* electronic transitions in these compounds facilitates transmission in the UV region; (2) High polymerized B–O groups effectively eliminate dangling bonds of O atoms in the anionic framework of three compounds. Notably, the UV cutoff edge of Li_2_Na_4_Ca_7_Sr_2_B_13_O_27_F_9_ is 241 nm, which is shorter than that of the other two compounds. This can be attributed to the high electronegativity of F.^55^

## Conclusions

In summary, two borates and one borate fluoride with highly symmetrical [B_12_O_24_] units were successfully synthesized via the high-temperature solution method. These three compounds exhibit anion groups ranging from three-dimensional to zero-dimensional structures. The correlation between the M/B ratio and structure dimension of compounds containing ring-like [B_12_O_24_] units was discussed. The LiNa_11_B_28_O_48_ compound contains a [B_15_O_30_] unit, while the Li_1.45_Na_7.55_B_21_O_36_ compound features a [B_21_O_36_] unit, both of which are novel FBBs with highly polymerized anion groups that enhance the structural diversity of borates. Besides, they exhibit thermal stability up to 700 °C and possess the ability of ultraviolet transmission. The exceptional thermal and optical properties render them advantageous for further applications.

## Supporting Information Available

Table of atomic coordinates, equivalent isotropic displacement parameters, bond valence sum, selected bond distances and angles, asymmetric unit, and atomic coordination environment of three compounds (PDF)
Crystallographic data 1 (CIF)
Crystallographic data 2 (CIF)
Crystallographic data 3 (CIF)

## Supplementary Material

ao3c02248_si_001.pdf

ao3c02248_si_002.cif

ao3c02248_si_003.cif

ao3c02248_si_004.cif

## Accession Codes

CCDC 2233774, 2233775, and 2233776 contain the supplementary crystallographic data for LiNa_11_B_28_O_48_, Li_1.45_Na_7.55_B_21_O_36_, and Li_2_Na_4_Ca_7_Sr_2_B_13_O_27_F_9_. The data can be obtained free of charge via www.ccdc.cam.ac.uk/data_request/cif, by emailing data_request@ccdc.cam.ac.uk, or by contacting The Cambridge Crystallographic Data Centre, 12 Union Road, Cambridge CB2 1EZ, U.K.; fax: + 44 1223 336033.

## References

[ref1] aBecker P. Borate Materials in Nonlinear Optics. Adv. Mater. 1998, 10, 979–992. 10.1002/(SICI)1521-4095(199809)10:13<979::AID-ADMA979>3.0.CO;2-N.

[ref2] aThompson R. Industrial Applications of Boron Compounds. Pure Appl. Chem. 1974, 39, 547–559. 10.1351/pac197439040547.

[ref3] Chen C. T.; Wu B. C.; Jiang A. D.; You G. M. A New Type Ultraviolet SHG Crystal: *β*-BaB_2_O_4_. Sci. Sin. B 1985, 28, 235–243.

[ref4] Chen C. T.; Wu Y. C.; Jiang A. D.; Wu B. C.; You G. M.; Li R. K.; Lin S. J. New Nonlinear-Optical Crystal: LiB_3_O_5_. J. Opt. Soc. Am. B 1989, 6, 616–621. 10.1364/JOSAB.6.000616.

[ref5] Chen C. T.; Wang Y. B.; Xia Y. N.; Wu B. C.; Tang D. Y.; Wu K. C.; Zeng W. R.; Yu L. H.; Mei L. F. New Development of Nonlinear Optical Crystals for the Ultraviolet Region with Molecular Engineering Approach. J. Appl. Phys. 1995, 77, 2268–2272. 10.1063/1.358814.

[ref6] Fang S. M. The Crystal Structure of Sodium Metaborate Na_3_(B_3_O_6_). Z. Krist.-Cryst. Mater. 1938, 99, 1–8. 10.1524/zkri.1938.99.1.1.

[ref7] Shi G. Q.; Wang Y.; Zhang F. F.; Zhang B. B.; Yang Z. H.; Hou X. L.; Pan S. L.; Poeppelmeier K. R. Finding the Next Deep-Ultraviolet Nonlinear Optical Material: NH_4_B_4_O_6_F. J. Am. Chem. Soc. 2017, 139, 10645–10648. 10.1021/jacs.7b05943. 28726399

[ref8] Zhang Z. Z.; Wang Y.; Zhang B. B.; Yang Z. H.; Pan S. L. Polar Fluorooxoborate, NaB_4_O_6_F: A Promising Material for Ionic Conduction and Nonlinear Optics. Angew. Chem., Int. Ed. 2018, 57, 6577–6581. 10.1002/anie.201803392. 29663612

[ref9] Wang Y.; Zhang B. B.; Yang Z. H.; Pan S. L. Cation-tuned Synthesis of Fluorooxoborates: Towards Optimal Deep-ultraviolet Nonlinear Optical Materials. Angew. Chem., Int. Ed. 2018, 57, 2150–2154. 10.1002/anie.201712168. 29316132

[ref10] Wang X. F.; Wang Y.; Zhang B. B.; Zhang F. F.; Yang Z. H.; Pan S. L. CsB_4_O_6_F: A Congruent-Melting Deep-Ultraviolet Nonlinear Optical Material by Combining Superior Functional Units. Angew. Chem., Int. Ed. 2017, 56, 14119–14123. 10.1002/anie.201708231. 28895656

[ref11] Mutailipu M.; Zhang M.; Zhang B. B.; Wang L. Y.; Yang Z. H.; Zhou X.; Pan S. L. SrB_5_O_7_F_3_ Functionalized with [B_5_O_9_F_3_]^6–^ Chromophores: Accelerating the Rational Design of Deep-Ultraviolet Nonlinear Optical Materials. Angew. Chem., Int. Ed. 2018, 57, 6095–6099. 10.1002/anie.201802058. 29498468

[ref12] Xia M.; Li F. M.; Mutailipu M.; Han S. J.; Yang Z. H.; Pan S. L. Discovery of First Magnesium Fluorooxoborate with Stable Fluorine Terminated Framework for Deep-UV Nonlinear Optical Application. Angew. Chem., Int. Ed. 2021, 60, 14650–14656. 10.1002/anie.202103657. 33871912

[ref13] Zhang Z. Z.; Wang Y.; Zhang B. B.; Yang Z. H.; Pan S. L. CaB_5_O_7_F_3_: A Beryllium-Free Alkaline-Earth Fluorooxoborate Exhibiting Excellent Nonlinear Optical Performances. Inorg. Chem. 2018, 57, 4820–4823. 10.1021/acs.inorgchem.8b00531. 29663805

[ref14] Zhang B. B.; Shi G. Q.; Yang Z. H.; Zhang F. F.; Pan S. L. Fluorooxoborates: Beryllium-Free Deep-Ultraviolet Nonlinear Optical Materials without Layered Growth. Angew. Chem., Int. Ed. 2017, 56, 3916–3919. 10.1002/anie.201700540. 28251767

[ref15] Shi G. Q.; Zhang F. F.; Zhang B. B.; Hou D. W.; Chen X. L.; Yang Z. H.; Pan S. L. Na_2_B_6_O_9_F_2_: A Fluoroborate with Short Cutoff Edge and Deep-Ultraviolet Birefringent Property Prepared by an Open High-Temperature Solution Method. Inorg. Chem. 2017, 56, 344–350. 10.1021/acs.inorgchem.6b02269. 27966921

[ref16] Chen Z. L.; Li Z. J.; Chu D. D.; Zhang F. F.; Li X. J.; Yang Z. H.; Long X. F.; Pan S. L. A_2_B_6_O_9_F_2_ (A = NH_4_, K): New Members of A_2_B_6_O_9_F_2_ Family with Deep-UV Cutoff Edges and Moderate Birefringence. Chem. Commun. 2022, 58, 12369–12372. 10.1039/D2CC04193F. 36263715

[ref17] Li X.; Chen Z. L.; Li F. M.; Zhang F. F.; Yang Z. H.; Pan S. L. LiNaB_6_O_9_F_2_: A Promising UV NLO Crystal Having Fluorine-Directed Optimal Performances and Double Interpenetrating ^3^[B_6_O_9_F_2_]_∞_ Networks. Adv. Optical Mater. 2023, 11, 2202195 10.1002/adom.202202195.

[ref18] Chen Z. L.; Feng J. W.; Dai B.; Yu F. NaKB_6_O_9_F_2_: A New Complex Alkali Metal Fluorooxoborate with 2 ∞[B_6_O_9_F_2_]^2-^ Puckered Layers. New J. Chem. 2021, 45, 2974–2980. 10.1039/D0NJ05124A.

[ref19] Han S. J.; Zhang B. B.; Yang Z. H.; Pan S. L. From LiB_3_O_5_ to NaRbB_6_O_9_F_2_: Fluorine-Directed Evolution of Structural Chemistry. Chem.–Eur. J. 2018, 24, 10022–10027. 10.1002/chem.201802466. 29863290

[ref20] Bubnova R. S.; Krivovichev S. V.; Shakhverdova I. P.; Filatov S. K.; Burns P. C.; Krzhizhanovskaya M.; Polyakova I. G. Synthesis, Crystal Structure and Thermal Behavior of Rb_3_B_7_O_12_, A New Compound. Solid State Sci. 2002, 4, 985–992. 10.1016/S1293-2558(02)01341-9.

[ref21] Krogh-Moe J.; Ihara M. On the Crystal Structure of Barium Tetraborate, BaO·4B_2_O_3_. Acta Cryst. B 1969, 25, 2153–2154. 10.1107/S0567740869005279.

[ref22] Penin N.; Touboul M.; Nowogrocki G. Refinement of α-CsB_9_O_14_ Crystal Structure. J. Solid State Chem. 2003, 175, 348–352. 10.1016/S0022-4596(03)00326-8.

[ref23] Liu L. L.; Su X.; Yang Y.; Pan S. L.; Dong X. Y.; Han S. J.; Zhang M.; Kang J.; Yang Z. H. Ba_2_B_10_O_17_: A New Centrosymmetric Alkaline-Earth Metal Borate with a Deep-UV Cut-Off Edge. Dalton Trans. 2014, 43, 8905–8910. 10.1039/C4DT00546E. 24801795

[ref24] Huang C. M.; Zhang F. F.; Zhang B. B.; Yang Z. H.; Pan S. L. NH_4_B_11_O_16_(OH)_2_: A New Ammonium Borate with Wavy-Shaped Polycyclic _∞_^2^[B_11_O_16_(OH)_2_] Layers. New J. Chem. 2018, 42, 12091–12097. 10.1039/C8NJ02359J.

[ref25] Jin C. C.; Li F. M.; Cheng B. L.; Qiu H. T.; Yang Z. H.; Pan S. L.; Mutailipu M. Double-Modification Oriented Design of a Deep-UV Birefringent Crystal Functionalized by [B_12_O_16_F_4_(OH)_4_] Clusters. Angew. Chem., Int. Ed. 2022, 61, e202203984 10.1002/anie.202203984. 35538644

[ref26] Zhang W. Y.; Wei Z. L.; Yang Z. H.; Pan S. L. Noncentrosymmetric Fluorooxoborates A_10_B_13_O_15_F_19_ (A = K and Rb) with Unexpected [B_10_O_12_F_13_]^7–^ Units and Deep-Ultraviolet Cutoff Edges. Inorg. Chem. 2020, 59, 3274–3280. 10.1021/acs.inorgchem.9b03707. 32037800

[ref27] Wu C. F.; Chen Z. L.; Chen J. B.; Yang Z. H.; Zhang F. F.; Shi H. S.; Pan S. L. Sr_3_B_14_O_24_: A New Borate with a [B_14_O_30_] Fundamental Building Block and an Unwonted 2D Double Layer. Dalton Trans. 2022, 51, 618–623. 10.1039/D1DT03653J. 34904978

[ref28] Merlino S.; Sartori F. Ammonioborite: New Borate Polyion and Its Structure. Science 1971, 171, 377–379. 10.1126/science.171.3969.377. 17808644

[ref29] Tang Z. H.; Chen X. A.; Li M. Synthesis and Crystal Structure of A New Strontium Borate, Sr_2_B_16_O_26_. Solid State Sci. 2008, 10, 894–900. 10.1016/j.solidstatesciences.2007.10.029.

[ref30] Yao W. J.; Jiang X. X.; Huang H. W.; Xu T.; Wang X. S.; Lin Z. S.; Chen C. T. Sr_8_MgB_18_O_36_: A New Alkaline-Earth Borate with A Novel Zero-Dimensional (B_18_O_36_)^18–^ Anion Ring. Inorg. Chem. 2013, 52, 8291–8293. 10.1021/ic401167z. 23848160

[ref31] Krzhizhanovskaya M. G.; Bannova I. I.; Filatov S. K.; Bubnova R. S. Crystal Structure and Thermal Expansion of Rb_5_B_19_O_31_. Crystallogr. Rep. 1999, 44, 187–192.

[ref32] Abudoureheman M.; Han S. J.; Wang Y.; Liu Q.; Yang Z. H.; Pan S. L. Three Mixed-Alkaline Borates: Na_2_M_2_B_20_O_32_ (M = Rb, Cs) with Two Interpenetrating Three-Dimensional B-O Networks and Li_4_Cs_4_B_40_O_64_ with Fundamental Building Block B_40_O_77_. Inorg. Chem. 2017, 56, 13456–13463. 10.1021/acs.inorgchem.7b02168. 28990767

[ref33] Mutailipu M.; Zhang M.; Su X.; Yang Z. H.; Pan S. L. Na_8_MB_21_O_36_ (M = Rb and Cs): Noncentrosymmetric Borates with Unprecedented [B_21_O_36_]^9–^ Fundamental Building Blocks. Inorg. Chem. 2017, 56, 5506–5509. 10.1021/acs.inorgchem.7b00671. 28436653

[ref34] Christ C. L.; Clark J. R. A Crystal-chemical Classification of Borate Structures with Emphasis on Hydrated Borates. Phys. Chem. Miner. 1977, 2, 59–87. 10.1007/BF00307525.

[ref35] Burns P. C.; Grice J. D.; Hawthorne F. C. Borate Minerals.I. Polyhedral Clusters and Fundamental Building Blocks. Can. Mineral. 1995, 33, 1131–1151.

[ref36] Menchetti S.; Sabelli C. A New Borate Polyanion in the Structure of Na_8_[B_12_O_20_(OH)_4_]. Acta Cryst. B 1979, 35, 2488–2493. 10.1107/S0567740879009742.

[ref37] Skakibaie-Moghadam M.; Heller G.; Timper U. Die Kristallstruktur von Ag_6_[B_12_O_18_(OH)_6_]·3H_2_O, einem neuen Dodekaborat. Z. Kristallogr. 1990, 190, 85–96. 10.1524/zkri.1990.190.1-2.85.

[ref38] Choudhury A.; Neeraj S.; Natarajan S.; Rao C. N. R. An Open-Framework Zincoborate Formed by Zn_6_B_12_O_24_ Clusters. J. Chem. Soc., Dalton Trans. 2002, 1535–1538. 10.1039/b108047b.

[ref39] Zhang H. X.; Zhang J.; Zheng S. T.; Yang G. Y. K_7_{(BO_3_)Mn[B_12_O_18_(OH)_6_]}·H_2_O: First Manganese Borate Based on Covalently Linked B_12_O_18_(OH)_6_ Clusters and BO_3_ Units via Mn^2+^ Cations. Inorg. Chem. Commun. 2004, 7, 781–783. 10.1016/j.inoche.2004.04.015.

[ref40] Rong C.; Jiang J.; Li Q. L. Synthesis of Transitional Metal Borate K_7_{(BO_3_)Zn[B_12_O_18_(OH)_6_]}·H_2_O and Quantum Chemistry Study. Chinese J. Inorg. Chem. 2012, 28, 2217–2222.

[ref41] Chen S. J.; Pan S. L.; Zhao W. W.; Yang Z. H.; Wu H. P.; Yang Y. Synthesis, Crystal Structure and Characterization of a New Compound, Li_3_NaBaB_6_O_12_. Solid State Sci. 2012, 14, 1186–1190. 10.1016/j.solidstatesciences.2012.05.026.

[ref42] Wu H. P.; Yu H. W.; Pan S. L.; Jiao A. Q.; Han J.; Wu K.; Han S. J.; Li H. Y. New Type of Complex Alkali and Alkaline Earth Metal Borates with Isolated (B_12_O_24_)^12–^ Anionic Group. Dalton Trans. 2014, 43, 4886–4891. 10.1039/c3dt53115e. 24492669

[ref43] Baiheti T.; Han S. J.; Bashir B.; Yang Z. H.; Wang Y.; Yu H. H.; Pan S. L. Four New Deep Ultraviolet Borates with Isolated B_12_O_24_ Groups: Synthesis, Structure, and Optical Properties. J. Solid State Chem. 2019, 273, 112–116. 10.1016/j.jssc.2019.02.034.

[ref44] Luo S. Y.; Yao W. J.; Gong P. F.; Yao J. Y.; Lin Z. S.; Chen C. T. Ca_3_Na_4_LiBe_4_B_10_O_24_F: A New Beryllium Borate with A Unique Beryl Borate ∞ 2[Be_8_B_16_O_40_F_2_] Layer Intrabridged by [B_12_O_24_] Groups. Inorg. Chem. 2014, 53, 8197–8199. 10.1021/ic501444t. 25084149

[ref45] Wang X. S.; Liu L. J.; Xia M. J.; Wang X. Y.; Chen C. T. Two Isostructural Multi-Metal Borates: Syntheses, Crystal Structures and Characterizations of M_3_LiNa_4_Be_4_B_10_O_24_F (M = Sr, Cd). Chinese J. Struct. Chem. 2015, 34, 1617–1625.

[ref46] Wang F. X.; Yang Y.; Jin C. C.; Pan S. L. Li_6.58_Na_7.43_Sr_4_(B_9_O_18_)(B_12_O_24_)Cl: Unprecedented Combination of the Largest Two Highly Polymerized Isolated B–O Clusters with Novel Isolated B_9_O_18_ FBB. Inorg. Chem. Front. 2022, 9, 4614–4623. 10.1039/D2QI01311H.

[ref47] Wei L.; Pan J.; Xue Z. Z.; Wang G. M.; Wang Y. X. A Novel Mixed-Metal Borate with Large [B_12_O_18_(OH)_6_]^6–^ Motif: Synthesis, Structure and Property. Solid State Sci. 2018, 75, 9–13. 10.1016/j.solidstatesciences.2017.11.004.

[ref48] Becker P. A Contribution to Borate Crystal Chemistry: Rules for the Occurrence of Polyborate Anion Types. Z. Kristallogr. - Cryst. Mater. 2001, 216, 523–533. 10.1524/zkri.216.10.523.20368.

[ref49] Leonyuk N. I. Structural Aspects in Crystal Growth of Anhydrous Borates. J. Cryst. Growth 1997, 174, 301–307. 10.1016/S0022-0248(96)01164-5.

[ref50] Sun J.; Lu X. Q.; Mutailipu M.; Pan S. L. Identical in Formula but not Isotypic in Configuration: Discovery of a new Highly Polymerized [B_12_O_24_] Cluster in Cs_3_AlB_6_O_12_. Inorg. Chem. 2021, 60, 15131–15135. 10.1021/acs.inorgchem.1c02593. 34591454

[ref51] Chen X. A.; Chen Y. J.; Sun C.; Chang X. N.; Xiao W. Q. Synthesis, Crystal Structure, Spectrum Properties, and Electronic Structure of a new Three-Borate Ba_4_Na_2_Zn_4_(B_3_O_6_)_2_(B_12_O_24_) with Two Isolated Types of Blocks: 3[3Δ] and 3[2Δ + 1T]. J. Alloys. Compd. 2013, 568, 60–67. 10.1016/j.jallcom.2013.03.103.

[ref52] MacDonald D. J.; Hawthorne F. C. The Crystal Chemistry of Si-Al Substitution in Tourmaline. Can. Mineral. 1995, 33, 849–858.

[ref53] Wang G. M.; Sun Y. Q.; Zheng S. T.; Yang G. Y. Synthesis and Crystal Structure of a Novel Potassium Borate with an Unprecedented [B_12_O_16_(OH)_8_]^4–^ Anion. Z. Anorg. Allg. Chem. 2006, 632, 1586–1590. 10.1002/zaac.200600054.

[ref54] aYan J. D.; Chu D. D.; Chen Z. L.; Han J. Li_2_PbB_2_O_5_: A Pyroborate with Large Birefringence Induced by the Synergistic Effect of Stereochemical Active Lone Pair Cations and π-Conjugated [B_2_O_5_] Groups. Inorg. Chem. 2022, 61, 18795–18801. 10.1021/acs.inorgchem.2c03469. 36331495

[ref55] aMutailipu M.; Zhang M.; Yang Z. H.; Pan S. L. Targeting the Next Generation of Deep-Ultraviolet Nonlinear Optical Materials: Expanding from Borates to Borate Fluorides to Fluorooxoborates. Acc. Chem. Res. 2019, 52, 791–801. 10.1021/acs.accounts.8b00649. 30794376

