# Supplementary material for: Three Polyborates with High-Symmetry [B12O24] Units Featuring Different Dimensions of Anion Groups
Source: ACS Omega. 2023 Jun 2;8(23):21172–81. doi: 10.1021/acsomega.3c02248 (PMC10268625; doi:10.1021/acsomega.3c02248)
Supplement: Supplementary file 1 — ao3c02248_si_001.pdf [file ao3c02248_si_001.pdf]

Electronic Supplementary Information

Three polyborates with high symmetry [$B_{12}O_{24}$] units featuring different dimensions of anion groups

Yu Dang,‡[a,b] Jingdong Yan,‡[a,b] Xueling Hou,*[a,b] and Hongsheng Shi*[a,b]

[a] *Research Center for Crystal Materials, CAS Key Laboratory of Functional Materials and Devices for Special Environments, Xinjiang Key Laboratory of Electronic Information Materials and Devices, Xinjiang Technical Institute of Physics & Chemistry, CAS,40-1 South Beijing Road, Urumqi 830011, China*

[b] *Center of Materials Science and Optoelectronics Engineering, University of Chinese Academy of Sciences, Beijing 100049, China*

‡ These authors contributed equally to this work.

Corresponding authors: xlhou@ms.xjb.ac.cn, shihs@ms.xjb.ac.cn

Table S1. Atomic coordinates (×10^4), equivalent isotropic displacement parameters (Å^2×10^3) and BVS for each atom in the asymmetric unit of $LiNa_{11}B_{28}O_{48}$. U_{eq} is defined as one-third of the trace of the orthogonalized U_{ij} tensor.

**Atoms**	**Wyck.**	**x**	**y**	**z**	U_{eq}	**BVS**
Li(1)	2b	0	0	5000	23(5)	1.06
Na(1)	12i	2783(3)	2747(3)	3881(1)	28(1)	0.72
Na(2)	4f	3333	6667	5013(2)	18(1)	0.94
Na(3)	6h	-123(5)	3231(5)	7500	32(1)	1.11
B(1)	12i	3100(7)	1511(7)	5001(2)	11(1)	3.03
B(2)	12i	3894(7)	3837(7)	5614(2)	10(1)	2.98
B(3)	12i	4011(7)	3969(7)	6554(2)	11(1)	3.06
B(4)	12i	1544(7)	1531(7)	6961(2)	8(1)	3.00
B(5)	6h	3656(13)	4162(13)	7500	25(2)	3.07
B(6)	2d	3333	6667	7500	30(4)	2.90
O(1)	12i	2129(4)	2082(5)	4737(1)	13(1)	2.02
O(2)	12i	4197(4)	2689(5)	5416(1)	13(1)	1.95
O(3)	12i	2794(5)	4187(5)	5408(1)	14(1)	1.97
O(4)	12i	4763(4)	4694(4)	6079(1)	12(1)	1.99
O(5)	12i	2565(5)	2565(5)	6518(1)	14(1)	1.91
O(6)	12i	4692(4)	4650(4)	7030(1)	13(1)	2.05
O(7)	12i	9(4)	1478(4)	6932(1)	10(1)	2.06
O(8)	6h	2447(6)	2389(6)	7500	9(1)	1.94
O(9)	6h	2690(8)	4999(8)	7500	29(2)	1.87

Table S2. Selected bond lengths [Å] for $LiNa_{11}B_{28}O_{48}$.

Li(1)-O(1)#1	2.106(4)	Na(3)-O(8)	2.925(7)
Li(1)-O(1)#4	2.106(4)	Na(3)-O(8)#4	2.690(7)
Li(1)-O(1)#5	2.106(4)	Na(3)-O(9)	2.340(8)
Li(1)-O(1)#3	2.106(4)	B(1)-O(1)	1.444(7)
Li(1)-O(1)	2.106(4)	B(1)-O(1)#3	1.457(7)
Li(1)-O(1)#2	2.106(4)	B(1)-O(2)	1.500(7)
Na(1)-O(1)	2.231(4)	B(1)-O(3)#3	1.497(7)
Na(1)-O(2)#1	2.851(5)	B(2)-O(2)	1.354(7)
Na(1)-O(3)#3	2.883(5)	B(2)-O(3)	1.348(7)
Na(1)-O(4)#6	2.385(4)	B(2)-O(4)	1.423(7)
Na(1)-O(5)#1	2.750(5)	B(3)-O(4)	1.381(7)
Na(1)-O(5)#3	2.703(5)	B(3)-O(5)	1.357(7)
Na(1)-O(7)#3	2.375(4)	B(3)-O(6)	1.354(7)
Na(2)-O(2)#1	2.365(4)	B(4)-O(5)	1.476(7)
Na(2)-O(2)#6	2.365(4)	B(4)-O(7)	1.435(7)
Na(2)-O(2)#7	2.365(4)	B(4)-O(7)#2	1.431(7)
Na(2)-O(3)	2.363(4)	B(4)-O(8)	1.587(6)
Na(2)-O(3)#8	2.363(4)	B(5)-O(6)	1.452(7)
Na(2)-O(3)#9	2.363(4)	B(5)-O(8)	1.491(11)
Na(3)-O(6)#12	2.265(5)	B(5)-O(9)	1.484(13)
Na(3)-O(6)#9	2.265(5)	B(6)-O(9)#8	1.384(7)
Na(3)-O(7)#10	2.239(5)	B(6)-O(9)#9	1.385(7)
Na(3)-O(7)	2.239(5)	B(6)-O(9)	1.384(7)

Table S3. Selected bond angles [deg] for $LiNa_{11}B_{28}O_{48}$.

O(1)#1-Li(1)-O(1)#5	110.73(9)	O(5)#1-Na(1)-O(3)#3	147.21(15)
O(1)#3-Li(1)-O(1)#4	180.0	O(5)#3-Na(1)-O(5)#1	101.43(17)
O(1)#4-Li(1)-O(1)#5	69.27(9)	O(7)#3-Na(1)-O(2)#1	122.39(16)
O(1)#2-Li(1)-O(1)#3	69.27(9)	O(7)#3-Na(1)-O(3)#3	122.66(15)
O(1)#1-Li(1)-O(1)#4	69.27(9)	O(7)#3-Na(1)-O(4)#6	123.47(16)
O(1)#4-Li(1)-O(1)	110.73(9)	O(7)#3-Na(1)-O(5)#3	54.70(12)
O(1)#5-Li(1)-O(1)	180.0	O(7)#3-Na(1)-O(5)#1	54.35(12)
O(1)#1-Li(1)-O(1)#2	180.0	O(2)#7-Na(2)-O(2)#6	101.08(15)
O(1)#2-Li(1)-O(1)#4	110.73(9)	O(2)#1-Na(2)-O(2)#6	101.08(15)
O(1)#3-Li(1)-O(1)#5	110.73(9)	O(2)#7-Na(2)-O(2)#1	101.08(15)
O(1)#1-Li(1)-O(1)	69.27(9)	O(3)#8-Na(2)-O(2)#7	98.04(13)
O(1)#3-Li(1)-O(1)	69.27(9)	O(3)-Na(2)-O(2)#1	60.96(12)
O(1)#1-Li(1)-O(1)#3	110.73(9)	O(3)#9-Na(2)-O(2)#6	156.11(13)
O(1)#2-Li(1)-O(1)#5	69.27(9)	O(3)#9-Na(2)-O(2)#1	98.04(13)
O(1)#2-Li(1)-O(1)	110.73(9)	O(3)#9-Na(2)-O(2)#7	60.96(12)
O(1)-Na(1)-O(2)#1	54.50(13)	O(3)#8-Na(2)-O(2)#6	60.96(12)
O(1)-Na(1)-O(3)#3	53.66(13)	O(3)-Na(2)-O(2)#6	98.04(13)
O(1)-Na(1)-O(4)#6	103.90(16)	O(3)#8-Na(2)-O(2)#1	156.11(13)
O(1)-Na(1)-O(5)#3	101.84(16)	O(3)-Na(2)-O(2)#7	156.11(13)
O(1)-Na(1)-O(5)#1	101.97(16)	O(3)#9-Na(2)-O(3)	103.73(15)
O(1)-Na(1)-O(7)#3	132.58(17)	O(3)#8-Na(2)-O(3)	103.73(15)
O(2)#1-Na(1)-O(3)#3	103.80(12)	O(3)#9-Na(2)-O(3)#8	103.73(15)
O(4)#6-Na(1)-O(2)#1	85.20(14)	O(6)#12-Na(3)-O(6)#9	62.5(2)
O(4)#6-Na(1)-O(3)#3	89.69(14)	O(6)#9-Na(3)-O(8)	124.67(19)
O(4)#6-Na(1)-O(5)#3	124.30(16)	O(6)#12-Na(3)-O(8)	124.67(19)
O(4)#6-Na(1)-O(5)#1	119.90(16)	O(6)#12-Na(3)-O(8)#4	129.5(2)
O(5)#3-Na(1)-O(2)#1	148.03(15)	O(6)#9-Na(3)-O(8)#4	129.5(2)
O(5)#1-Na(1)-O(2)#1	68.07(12)	O(6)#12-Na(3)-O(9)	81.1(2)
O(5)#3-Na(1)-O(3)#3	68.04(12)	O(6)#9-Na(3)-O(9)	81.1(2)
O(7)#10-Na(3)-O(6)#12	109.22(13)	O(7)-B(4)-O(8)	109.8(4)

O(7)#10-Na(3)-O(6)#9	170.72(19)	O(3)-B(2)-O(2)	124.2(5)
O(7)-Na(3)-O(6)#12	170.72(19)	O(3)-B(2)-O(4)	118.1(5)
O(7)-Na(3)-O(6)#9	109.22(13)	O(5)-B(3)-O(4)	116.8(5)
O(7)-Na(3)-O(7)#10	78.7(2)	O(6)-B(3)-O(4)	120.9(5)
O(7)#10-Na(3)-O(8)	55.37(14)	O(6)-B(3)-O(5)	122.4(5)
O(7)-Na(3)-O(8)	55.37(14)	O(5)-B(4)-O(8)	106.8(4)
O(7)-Na(3)-O(8)#4	58.49(14)	O(7)-B(4)-O(5)	107.7(4)
O(7)#10-Na(3)-O(8)#4	58.49(14)	O(7)#2-B(4)-O(5)	108.9(4)
O(7)-Na(3)-O(9)	93.7(2)	O(7)#2-B(4)-O(7)	115.5(5)
O(7)#10-Na(3)-O(9)	93.7(2)	O(7)-B(4)-O(8)	109.8(4)
O(8)#4-Na(3)-O(8)	90.2(2)	O(7)#2-B(4)-O(8)	107.8(4)
O(9)-Na(3)-O(8)#4	142.3(3)	O(6)-B(5)-O(6)#10	108.1(7)
O(9)-Na(3)-O(8)	52.1(2)	O(6)#10-B(5)-O(8)	112.2(5)
O(1)-B(1)-O(1)#3	111.3(5)	O(6)-B(5)-O(8)	112.2(5)
O(1)-B(1)-O(2)	111.8(5)	O(6)-B(5)-O(9)	109.3(6)
O(1)#3-B(1)-O(2)	108.0(4)	O(6)#10-B(5)-O(9)	109.3(6)
O(1)#3-B(1)-O(3)#3	111.3(5)	O(9)-B(5)-O(8)	105.8(7)
O(1)-B(1)-O(3)#3	108.1(4)	O(9)#9-B(6)-O(9)	120.000(4)
O(3)#3-B(1)-O(2)	106.3(4)	O(9)#8-B(6)-O(9)	120.001(2)
O(2)-B(2)-O(4)	117.7(5)	O(9)#9-B(6)-O(9)#8	119.998(5)

Symmetry transformations used to generate equivalent atoms:

#1 x-y,x,-z+1 **#2 -x+y,-x,z** **#3 y,-x+y,-z+1**

#4 -y,x-y,z **#5 -x,-y,-z+1** **#6 -x+1,-y+1,-z+1**

#7 y,-x+y+1,-z+1 **#8 -y+1,x-y+1,z** **#9 -x+y,-x+1,z**

#10 x,y,-z+3/2 **#11 -y,x-y,-z+3/2** **#12 -x+y,-x+1,-z+3/2**

Table S4. Atomic coordinates (×10^4), equivalent isotropic displacement parameters (Å^2×10^3) and BVS for each atom in the asymmetric unit of $Li_{1.45}Na_{7.55}B_{21}O_{36}$. U_{eq} is defined as one-third of the trace of the orthogonalized U_{ij} tensor.

**Atoms**	**Wyck.**	**x**	**y**	**z**	**U_{eq}**	**BVS**
Li/Na(1)	4d	3333	6667	5000	18(1)	1.23
Li(2)	2b	0	10000	5000	24(2)	1.08
Na(2)	12k	2706(1)	10000	6432(1)	26(1)	0.99
B(1)	12i	1570(1)	8430(1)	5000	17(1)	3.08
B(2)	6g	0	8423(3)	7500	12(1)	3.03
B(3)	12k	0	6101(2)	5760(1)	15(1)	3.03
B(4)	12k	0	6018(2)	6917(1)	16(1)	3.05
O(1)	24l	1469(1)	7232(1)	5510(1)	20(1)	2.11
O(2)	12k	2133(2)	10000	5329(1)	18(1)	2.07
O(3)	6g	1498(2)	10000	7500	16(1)	2.03
O(4)	12k	0	7462(2)	6897(1)	18(1)	1.95
O(5)	12k	0	5249(1)	6341(1)	16(1)	2.10
O(6)	6g	0	5256(2)	7500	24(1)	1.97

Table S5. Selected bond lengths [Å] for $Li_{1.45}Na_{7.55}B_{21}O_{36}$.

Li/Na(1)-O(1)#1	2.3006(10)	Na(2)-O(4)#7	2.6272(9)
Li/Na(1)-O(1)#2	2.3006(10)	Na(2)-O(5)#2	2.3838(16)
Li/Na(1)-O(1)	2.3006(10)	B(1)-O(1)#1	1.4883(16)
Li/Na(1)-O(1)#3	2.3006(10)	B(1)-O(1)	1.4884(16)
Li/Na(1)-O(1)#4	2.3006(10)	B(1)-O(2)#6	1.4492(14)
Li/Na(1)-O(1)#5	2.3006(10)	B(1)-O(2)	1.4492(14)
Li(2)-O(2)#7	2.1011(15)	B(2)-O(3)	1.4385(17)
Li(2)-O(2)#9	2.1011(15)	B(2)-O(3)#8	1.4385(17)
Li(2)-O(2)#6	2.1011(15)	B(2)-O(4)#13	1.514(2)
Li(2)-O(2)	2.1011(15)	B(2)-O(4)	1.514(2)
Li(2)-O(2)#8	2.1011(15)	B(3)-O(1)	1.3440(14)
Li(2)-O(2)#10	2.1011(15)	B(3)-O(1)#14	1.3440(14)
Na(2)-O(1)#11	2.9171(12)	B(3)-O(5)	1.418(2)
Na(2)-O(1)	2.9171(12)	B(4)-O(4)	1.350(2)
Na(2)-O(2)	2.2913(15)	B(4)-O(5)	1.368(2)
Na(2)-O(3)	2.4349(12)	B(4)-O(6)	1.377(2)
Na(2)-O(4)	2.6272(9)		

Table S6. Selected bond angles [deg] for $Li_{1.45}Na_{7.55}B_{21}O_{36}$.

O(1)#1-Li/Na(1)-O(1)	62.57(5)	O(2)-Na(2)-O(1)	52.40(2)
O(1)#4-Li/Na(1)-O(1)#3	101.53(3)	O(2)-Na(2)-O(1)#11	52.40(2)
O(1)#4-Li/Na(1)-O(1)#5	62.57(5)	O(2)-Na(2)-O(3)	138.86(7)
O(1)#2-Li/Na(1)-O(1)#3	62.57(5)	O(2)-Na(2)-O(4)	103.17(4)
O(1)-Li/Na(1)-O(1)#5	101.53(3)	O(2)-Na(2)-O(4)#7	103.17(4)
O(1)#2-Li/Na(1)-O(1)#5	101.53(3)	O(2)-Na(2)-O(5)#2	99.08(6)
O(1)#1-Li/Na(1)-O(1)#4	101.53(3)	O(3)-Na(2)-O(1)	126.01(3)
O(1)#1-Li/Na(1)-O(1)#2	98.06(5)	O(3)-Na(2)-O(1)#11	126.01(3)
O(1)-Li/Na(1)-O(1)#4	98.06(5)	O(3)-Na(2)-O(4)	56.39(4)
O(1)#1-Li/Na(1)-O(1)#5	157.05(5)	O(3)-Na(2)-O(4)#7	56.39(4)
O(1)#2-Li/Na(1)-O(1)#4	157.05(5)	O(4)#7-Na(2)-O(1)#11	69.64(4)
O(1)-Li/Na(1)-O(1)#2	101.53(3)	O(4)-Na(2)-O(1)	69.64(4)
O(1)#1-Li/Na(1)-O(1)#3	101.53(3)	O(4)-Na(2)-O(1)#11	148.49(5)
O(1)-Li/Na(1)-O(1)#3	157.05(5)	O(4)#7-Na(2)-O(1)	148.50(5)
O(1)#3-Li/Na(1)-O(1)#5	98.06(5)	O(4)-Na(2)-O(4)#7	102.84(6)
O(4)#10-Li(2)-O(4)#9	110.47(3)	O(5)#2-Na(2)-O(1)	84.46(3)
O(4)#10-Li(2)-O(4)#8	180.0	O(5)#2-Na(2)-O(1)#11	84.46(3)
O(4)#6-Li(2)-O(4)	69.53(3)	O(5)#2-Na(2)-O(3)	122.06(6)
O(4)#9-Li(2)-O(4)	180.0	O(5)#2-Na(2)-O(4)	122.50(3)
O(4)#8-Li(2)-O(4)#6	69.53(3)	O(5)#2-Na(2)-O(4)#7	122.50(4)
O(4)#8-Li(2)-O(4)#7	110.47(3)	O(1)#1-B(1)-O(1)	106.78(15)
O(4)#7-Li(2)-O(4)#9	69.53(3)	O(2)#6-B(1)-O(1)#1	107.37(6)
O(4)#8-Li(2)-O(4)#9	69.53(3)	O(2)#6-B(1)-O(1)	111.88(6)
O(4)#10-Li(2)-O(4)	69.53(3)	O(2)-B(1)-O(1)#1	111.87(6)
O(4)#10-Li(2)-O(4)#6	110.47(3)	O(2)-B(1)-O(1)	107.37(6)
O(4)#6-Li(2)-O(4)#7	180.00(6)	O(2)-B(1)-O(2)#6	111.52(19)
O(4)#6-Li(2)-O(4)#9	110.47(3)	O(3)-B(2)-O(3)#8	114.9(2)
O(4)#10-Li(2)-O(4)#7	69.53(3)	O(3)-B(2)-O(4)#13	108.60(4)
O(4)#7-Li(2)-O(4)	110.47(3)	O(3)#8-B(2)-O(4)	108.60(4)
O(1)-Na(2)-O(1)#11	100.33(4)	O(4)#13-B(2)-O(4)	107.3(2)

O(3)-B(2)-O(4)	108.60(4)	O(1)-B(3)-O(1)#14	124.43(17)
O(3)#8-B(2)-O(4)#13	108.60(4)	O(1)#14-B(3)-O(5)	117.77(9)
O(1)-B(3)-O(5)	117.76(9)	O(4)-B(4)-O(6)	122.85(18)
O(4)-B(4)-O(5)	119.96(17)	O(5)-B(4)-O(6)	117.19(18)

Symmetry transformations used to generate equivalent atoms:

#1 -y+1,-x+1,-z+1 **#2 -x+y,-x+1,z** **#3 -x+y,y,-z+1**

#4 x,x-y+1,-z+1 **#5 -y+1,x-y+1,z** **#6 y-1,-x+y,-z+1**

#7 -y+1,x-y+2,z **#8 -x+y-1,-x+1,z** **#9 -x,-y+2,-z+1**

#10 x-y+1,x+1,-z+1 **#11 x-y+1,-y+2,z** **#12 -x+y-1,-x+1,-z+3/2**

#13 x,y,-z+3/2 **#14 -x,-x+y,z**

Table S7. Atomic coordinates (×10^4), equivalent isotropic displacement parameters (Å^2×10^3) and BVS for each atom in the asymmetric unit of $Li_2Na_4Ca_7Sr_2B_{13}O_{27}F_9$. U_{eq} is defined as one-third of the trace of the orthogonalized U_{ij} tensor.

**Atoms**	**Wyck.**	***x***	***y***	***z***	**U_{eq}**	**BVS**
Li(1)	2a	0	10000	2500	25(3)	0.81
Li(2)	2b	0	0	5000	36(4)	1.07
Na/Li(1)	4f	-3333	3333	4731(1)	26(1)	1.15
Na(2)	4f	3333	6667	3671(2)	28(1)	1.03
Ca/Sr(1)	12i	-295(1)	7045(1)	3473(1)	12(1)	1.89
Ca(2)	6h	148(1)	4044(1)	2500	8(1)	1.88
B(1)	2d	-3333	3333	2500	19(2)	2.72
B(2)	12i	216(4)	4039(4)	4183(2)	15(1)	3.00
B(3)	12i	1625(4)	3164(4)	5031(2)	15(1)	3.07
O(1)	6h	-2848(4)	2128(4)	2500	23(1)	1.78
O(2)	12i	363(3)	4962(3)	3639(1)	20(1)	2.01
O(3)	12i	-1319(3)	2752(3)	4391(1)	18(1)	2.08
O(4)	12i	1621(3)	4366(3)	4550(1)	19(1)	2.05
O(5)	12i	2135(3)	2140(3)	4660(1)	18(1)	2.04
F(1)	6h	2895(3)	4466(4)	2500	28(1)	0.75
F(2)	12i	-1823(3)	8318(2)	3173(1)	31(1)	0.80

Table S8. Selected bond lengths [Å] for $Li_2Na_4Ca_7Sr_2B_{13}O_{27}F_9$.

Li(1)-F(2)	2.104(2)	Ca/Sr(1)-O(1)#14	2.625(2)
Li(1)-F(2)#2	2.104(2)	Ca/Sr(1)-O(2)	2.336(2)
Li(1)-F(2)#1	2.104(2)	Ca/Sr(1)-O(3)#14	2.681(2)
Li(1)-F(2)#3	2.104(2)	Ca/Sr(1)-O(4)#16	3.243(2)
Li(1)-F(2)#4	2.104(2)	Ca/Sr(1)-O(5)#16	2.419(2)
Li(1)-F(2)#5	2.104(2)	Ca/Sr(1)-F(1)#16	2.565(2)
Li(2)-O(5)#9	2.103(2)	Ca/Sr(1)-F(2)	2.341(2)
Li(2)-O(5)	2.103(2)	Ca/Sr(1)-F(2)#2	2.408(2)
Li(2)-O(5)#7	2.103(2)	Ca(2)-O(1)	2.452(3)
Li(2)-O(5)#8	2.103(2)	Ca(2)-O(1)#14	2.700(3)
Li(2)-O(5)#6	2.103(2)	Ca(2)-O(2)#1	2.364(2)
Li(2)-O(5)#10	2.103(2)	Ca(2)-O(2)	2.364(2)
Na(1)-O(3)	2.300(2)	Ca(2)-F(1)#16	2.476(3)
Na(1)-O(3)#12	2.300(2)	Ca(2)-F(1)	2.390(3)
Na(1)-O(3)#14	2.300(2)	Ca(2)-F(2)#17	2.453(2)
Na(1)-O(4)#13	2.391(3)	Ca(2)-F(2)#12	2.453(2)
Na(1)-O(4)#9	2.391(3)	B(1)-O(1)#12	1.407(3)
Na(1)-O(4)#11	2.391(3)	B(1)-O(1)#14	1.407(3)
Na(2)-O(2)#16	2.409(2)	B(1)-O(1)	1.407(3)
Na(2)-O(2)#15	2.409(2)	B(2)-O(2)	1.332(4)
Na(2)-O(2)	2.409(2)	B(2)-O(3)	1.393(4)
Na(2)-O(4)#16	2.588(3)	B(2)-O(4)	1.389(4)
Na(2)-O(4)	2.588(3)	B(3)-O(3)#7	1.488(4)
Na(2)-O(4)#15	2.588(3)	B(3)-O(4)	1.467(4)
Na(2)-F(1)#15	2.968(3)	B(3)-O(5)	1.458(4)
Na(2)-F(1)	2.968(3)	B(3)-O(5)#9	1.463(4)
Na(2)-F(1)#16	2.968(3)		

Table S9. Selected bond angles [deg] for $Li_2Na_4Ca_7Sr_2B_{13}O_{27}F_9$.

F(2)#3-Li(1)-F(2)#4	84.88(8)	O(5)#9-Li(2)-O(5)	69.59(5)
F(2)#3-Li(1)-F(2)	134.14(4)	O(5)#8-Li(2)-O(5)	180.0
F(2)#1-Li(1)-F(2)#5	134.14(4)	O(3)-Na(1)-O(3)#14	111.96(7)
F(2)#1-Li(1)-F(2)#3	84.88(8)	O(3)-Na(1)-O(3)#12	111.97(7)
F(2)#4-Li(1)-F(2)	134.14(4)	O(3)#12-Na(1)-O(3)#14	111.97(7)
F(2)#2-Li(1)-F(2)#4	77.63(11)	O(3)-Na(1)-O(4)#13	149.58(10)
F(2)#1-Li(1)-F(2)	77.63(12)	O(3)#14-Na(1)-O(4)#13	97.15(8)
F(2)#5-Li(1)-F(2)#2	84.88(8)	O(3)#12-Na(1)-O(4)#9	97.15(8)
F(2)#5-Li(1)-F(2)#3	77.63(11)	O(3)#12-Na(1)-O(4)#11	149.58(10)
F(2)#2-Li(1)-F(2)#3	134.14(4)	O(3)#14-Na(1)-O(4)#11	61.68(7)
F(2)#5-Li(1)-F(2)	84.87(8)	O(3)#12-Na(1)-O(4)#13	61.68(7)
F(2)#1-Li(1)-F(2)#4	84.88(8)	O(3)#14-Na(1)-O(4)#9	149.58(10)
F(2)#1-Li(1)-F(2)#2	134.14(4)	O(3)-Na(1)-O(4)#11	97.15(8)
F(2)#2-Li(1)-F(2)	84.88(8)	O(3)-Na(1)-O(4)#9	61.68(7)
F(2)#5-Li(1)-F(2)#4	134.14(4)	O(4)#11-Na(1)-O(4)#13	88.79(10)
F(2)#3-Li(1)-F(2)#4	84.88(8)	O(4)#9-Na(1)-O(4)#11	88.79(10)
O(5)#9-Li(2)-O(5)#10	180.0	O(4)#9-Na(1)-O(4)#13	88.79(10)
O(5)#10-Li(2)-O(5)	110.41(5)	O(2)#16-Na(2)-O(2)#15	119.935(9)
O(5)#7-Li(2)-O(5)#8	110.41(5)	O(2)-Na(2)-O(2)#15	119.935(8)
O(5)#6-Li(2)-O(5)#9	69.59(5)	O(2)-Na(2)-O(2)#16	119.935(8)
O(5)#7-Li(2)-O(5)#6	180.0	O(2)#15-Na(2)-O(4)#16	135.65(13)
O(5)#8-Li(2)-O(5)#10	69.59(5)	O(2)-Na(2)-O(4)#15	135.65(13)
O(5)#7-Li(2)-O(5)	69.59(5)	O(2)#16-Na(2)-O(4)#15	84.02(8)
O(5)#7-Li(2)-O(5)#9	110.41(6)	O(2)#15-Na(2)-O(4)#15	55.96(7)
O(5)#8-Li(2)-O(5)#9	110.41(5)	O(2)-Na(2)-O(4)	55.95(7)
O(5)#7-Li(2)-O(5)#10	69.59(5)	O(2)#15-Na(2)-O(4)	84.02(8)
O(5)#6-Li(2)-O(5)	110.41(5)	O(2)#16-Na(2)-O(4)	135.65(13)
O(5)#6-Li(2)-O(5)#10	110.41(6)	O(2)#16-Na(2)-O(4)#16	55.96(7)
O(5)#6-Li(2)-O(5)#8	69.59(6)	O(2)-Na(2)-O(4)#16	84.02(8)
O(2)-Na(2)-F(1)#16	62.47(8)	O(2)-Na(2)-F(1)#16	62.47(8)

O(2)#16-Na(2)-F(1)#16	78.94(9)	O(2)-Ca/Sr(1)-F(2)	159.59(8)
O(2)-Na(2)-F(1)	78.94(9)	O(3)#14-Ca/Sr(1)-O(4)#16	97.27(6)
O(2)#15-Na(2)-F(1)#16	126.49(13)	O(5)#16-Ca/Sr(1)-O(1)#14	148.88(8)
O(2)#15-Na(2)-F(1)#15	78.94(9)	O(5)#16-Ca/Sr(1)-O(3)#14	54.52(7)
O(2)#16-Na(2)-F(1)#15	62.47(8)	O(5)#16-Ca/Sr(1)-O(4)#16	46.59(6)
O(2)#16-Na(2)-F(1)	126.49(13)	O(5)#16-Ca/Sr(1)-F(1)#16	137.55(8)
O(2)#15-Na(2)-F(1)	62.47(8)	F(1)#16-Ca/Sr(1)-O(1)#14	70.52(8)
O(2)-Na(2)-F(1)#15	126.49(13)	F(1)#16-Ca/Sr(1)-O(3)#14	161.25(8)
O(4)#16-Na(2)-O(4)	80.52(10)	F(1)#16-Ca/Sr(1)-O(4)#16	91.23(7)
O(4)#16-Na(2)-O(4)#15	80.52(10)	F(2)#2-Ca/Sr(1)-O(1)#14	118.28(8)
O(4)#15-Na(2)-O(4)	80.52(10)	F(2)-Ca/Sr(1)-O(1)#14	80.69(9)
O(4)#16-Na(2)-F(1)#16	97.27(7)	F(2)-Ca/Sr(1)-O(3)#14	75.32(7)
O(4)#15-Na(2)-F(1)#16	160.61(8)	F(2)#2-Ca/Sr(1)-O(3)#14	128.88(7)
O(4)-Na(2)-F(1)	97.27(7)	F(2)-Ca/Sr(1)-O(4)#16	127.34(7)
O(4)#15-Na(2)-F(1)	118.30(7)	F(2)#2-Ca/Sr(1)-O(4)#16	72.21(7)
O(4)-Na(2)-F(1)#16	118.31(7)	F(2)#2-Ca/Sr(1)-O(5)#16	89.19(7)
O(4)#15-Na(2)-F(1)#15	97.27(7)	F(2)-Ca/Sr(1)-O(5)#16	94.87(8)
O(4)#16-Na(2)-F(1)#15	118.31(7)	F(2)-Ca/Sr(1)-F(1)#16	112.38(8)
O(4)-Na(2)-F(1)#15	160.61(8)	F(2)#2-Ca/Sr(1)-F(1)#16	69.66(8)
O(4)#16-Na(2)-F(1)	160.61(8)	F(2)-Ca/Sr(1)-F(2)#2	73.40(10)
F(1)#16-Na(2)-F(1)	66.63(9)	O(1)-Ca(2)-O(1)#14	56.22(14)
F(1)#16-Na(2)-F(1)#15	66.63(9)	O(1)-Ca(2)-F(1)#16	126.82(11)
F(1)#15-Na(2)-F(1)	66.63(9)	O(1)-Ca(2)-F(2)#17	82.16(8)
O(1)#14-Ca/Sr(1)-O(3)#14	94.89(7)	O(1)-Ca(2)-F(2)#12	82.16(8)
O(1)#14-Ca/Sr(1)-O(4)#16	151.52(8)	O(2)-Ca(2)-O(1)#14	79.38(6)
O(2)-Ca/Sr(1)-O(1)#14	81.46(9)	O(2)#1-Ca(2)-O(1)#14	79.38(6)
O(2)-Ca/Sr(1)-O(3)#14	96.48(7)	O(2)-Ca(2)-O(1)	97.08(6)
O(2)-Ca/Sr(1)-O(4)#16	71.66(7)	O(2)#1-Ca(2)-O(1)	97.08(6)
O(2)-Ca/Sr(1)-O(5)#16	95.30(8)	O(2)#1-Ca(2)-O(2)	141.68(11)
O(2)-Ca/Sr(1)-F(1)#16	70.34(8)	O(2)#1-Ca(2)-F(1)#16	71.49(6)
O(2)-Ca/Sr(1)-F(2)#2	124.37(8)	F(2)#17-Ca(2)-F(1)#16	138.41(7)

O(2)-Ca(2)-F(1)	92.90(6)	F(2)#12-Ca(2)-F(1)#16	138.41(7)
O(2)-Ca(2)-F(1)#16	71.49(6)	F(2)#17-Ca(2)-F(2)#12	65.04(11)
O(2)#1-Ca(2)-F(1)	92.90(6)	O(1)#14-B(1)-O(1)#12	120.0
O(2)-Ca(2)-F(2)#12	76.24(8)	O(1)-B(1)-O(1)#14	120.002(1)
O(2)#1-Ca(2)-F(2)#17	76.24(7)	O(1)-B(1)-O(1)#12	120.001(1)
O(2)-Ca(2)-F(2)#17	141.05(8)	O(2)-B(2)-O(3)	121.4(3)
O(2)#1-Ca(2)-F(2)#12	141.05(8)	O(2)-B(2)-O(4)	119.4(3)
F(1)-Ca(2)-O(1)#14	154.72(10)	O(4)-B(2)-O(3)	119.2(3)
F(1)-Ca(2)-O(1)	149.06(11)	O(4)-B(3)-O(3)#7	109.0(2)
F(1)#16-Ca(2)-O(1)#14	70.60(10)	O(5)#9-B(3)-O(3)#7	105.4(2)
F(1)-Ca(2)-F(1)#16	84.12(15)	O(5)-B(3)-O(3)#7	112.6(2)
F(1)-Ca(2)-F(2)#17	71.90(8)	O(5)#9-B(3)-O(4)	111.7(2)
F(1)-Ca(2)-F(2)#12	71.90(8)	O(5)-B(3)-O(4)	107.8(2)
F(2)#17-Ca(2)-O(1)#14	128.01(7)	O(5)-B(3)-O(5)#9	110.5(3)

Symmetry transformations used to generate equivalent atoms:

#1 x,y,-z+1/2 **#2 -y+1,x-y+2,z** **#3 -x+y-1,-x+1,-z+1/2**
#4 -y+1,x-y+2,-z+1/2 **#5 -x+y-1,-x+1,z** **#6 -y,x-y,z**
#7 y,-x+y,-z+1 **#8 -x,-y,-z+1** **#9 x-y,x,-z+1**
#10 -x+y,-x,z **#11 -x,-y+1,-z+1** **#12 -x+y-1,-x,z**
#13 y-1,-x+y,-z+1 **#14 -y,x-y+1,z** **#15 -y+1,x-y+1,z**
#16 -x+y,-x+1,z **#17 -x+y-1,-x,-z+1/2** **#18 -y+1,x-y+1,-z+1/2**

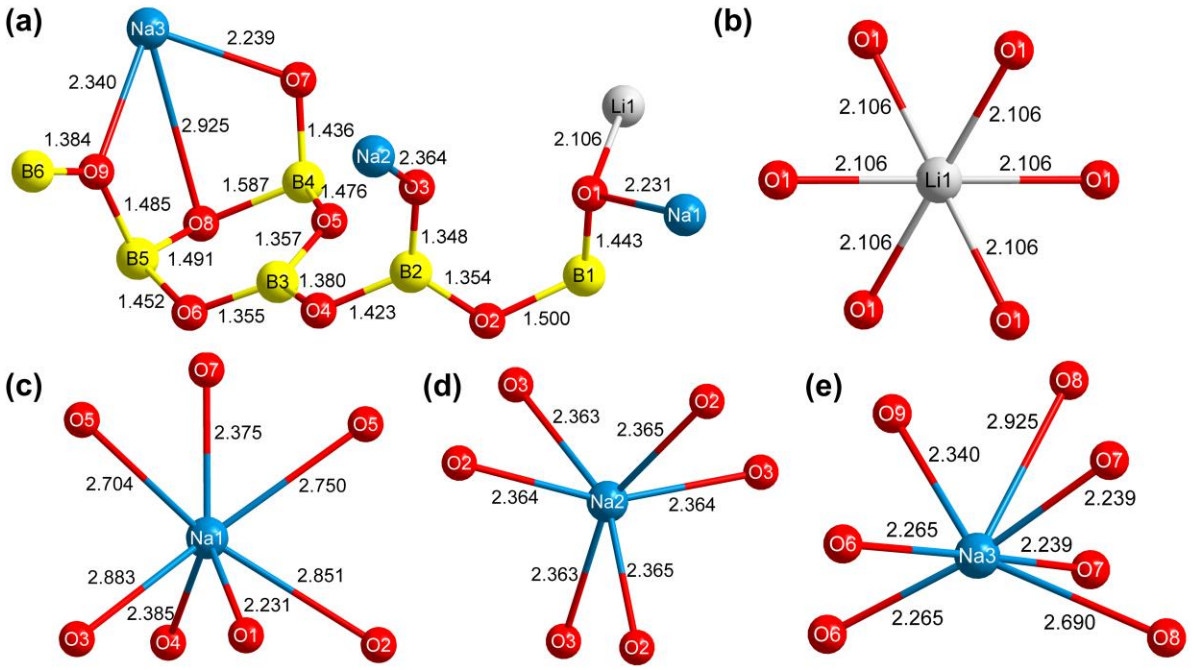

Figure S1. The asymmetric unit of $LiNa_{11}B_{28}O_{48}$ (a), and cation coordination environments of $[Li(1)O_6]$ (b), $[Na(1)O_7]$ (c), $[Na(2)O_6]$ (d) and $[Na(3)O_7]$ (e) in $LiNa_{11}B_{28}O_{48}$.

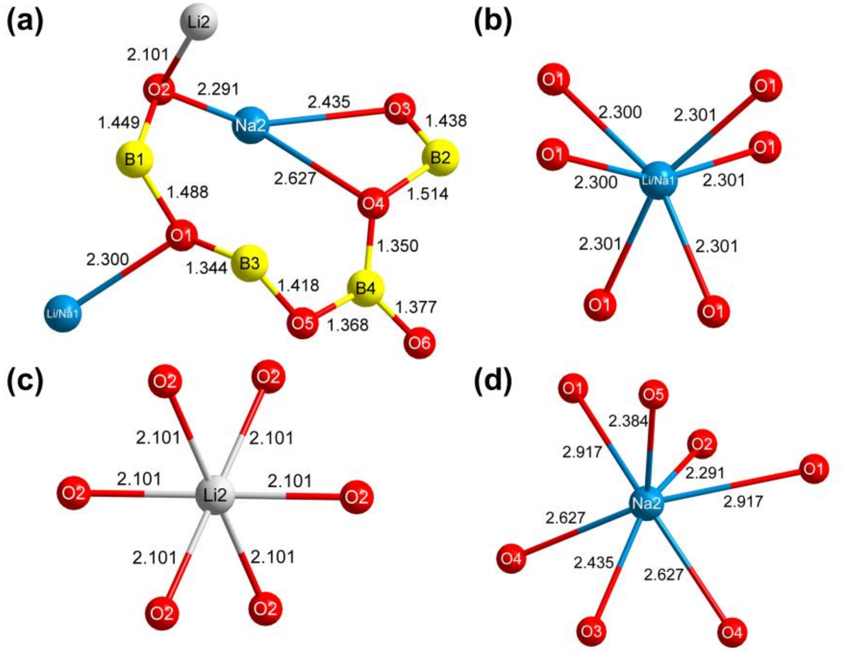

Figure S2. The asymmetric unit of $Li_{1.45}Na_{7.55}B_{21}O_{36}$ (a), and cation coordination environments of [Li/Na(1)O_6] (b), [Li(2)O_6] (c) and [Na(2)O_7] (d) in $Li_{1.45}Na_{7.55}B_{21}O_{36}$.

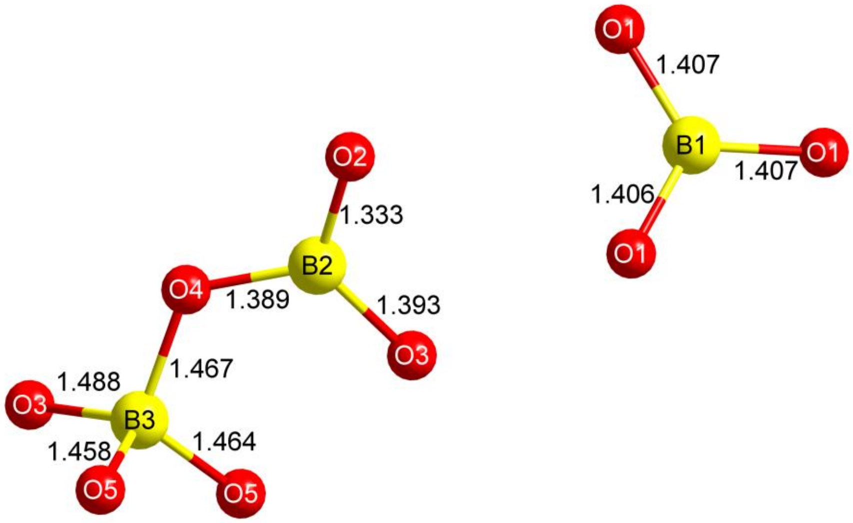

Figure S3. The anion coordination environments of $Li_2Na_4Ca_7Sr_2B_{13}O_{27}F_9$.

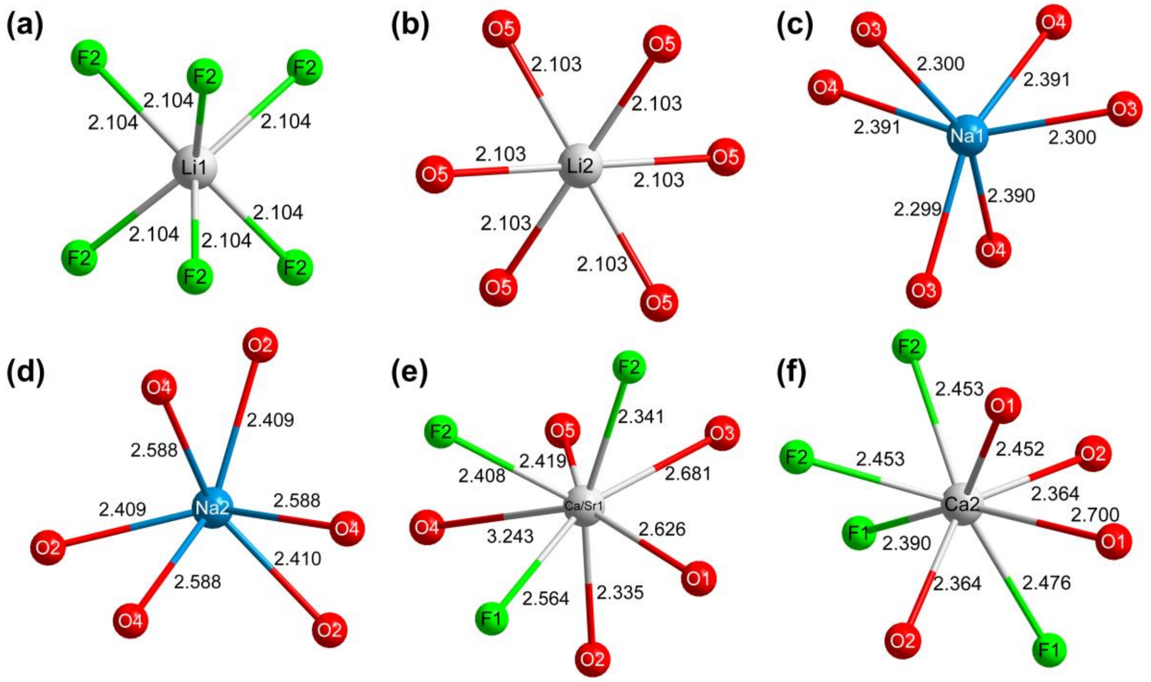

Figure S4. The cation coordination environments of $[Li(1)F_6]$ (a), $[Li(2)O_6]$ (b), $[Na(1)O_6]$ (c), $[Na(2)O_6]$ (d), $[Ca/Sr(1)O_5F_3]$ (e) and $[Ca(2)O_4F_4]$ (f) in $Li_{1.45}Na_{7.55}B_{21}O_{36}$.

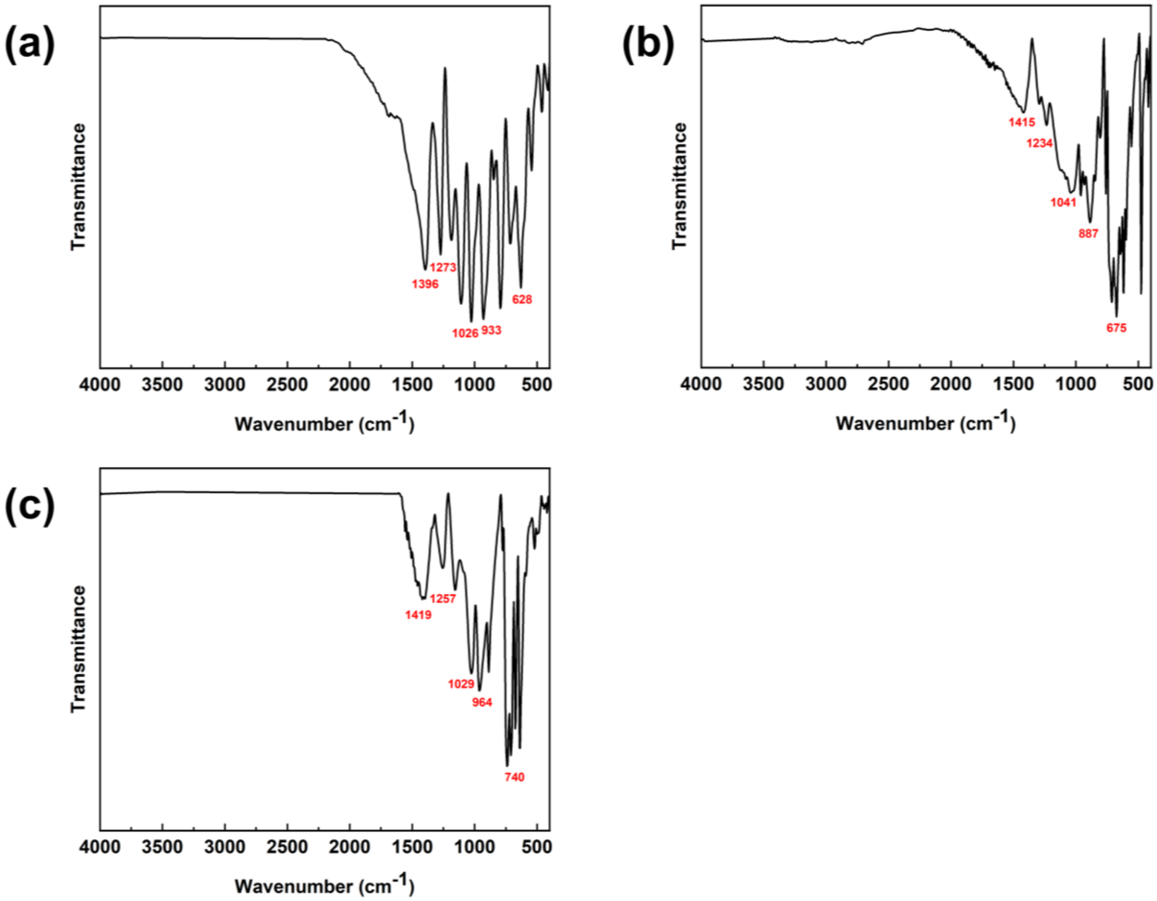

Figure S5. The IR spectrum of $LiNa_{11}B_{28}O_{48}$ (a), $Li_{1.45}Na_{7.55}B_{21}O_{36}$ (b), $Li_2Na_4Ca_7Sr_2B_{13}O_{27}F_9$ (c).

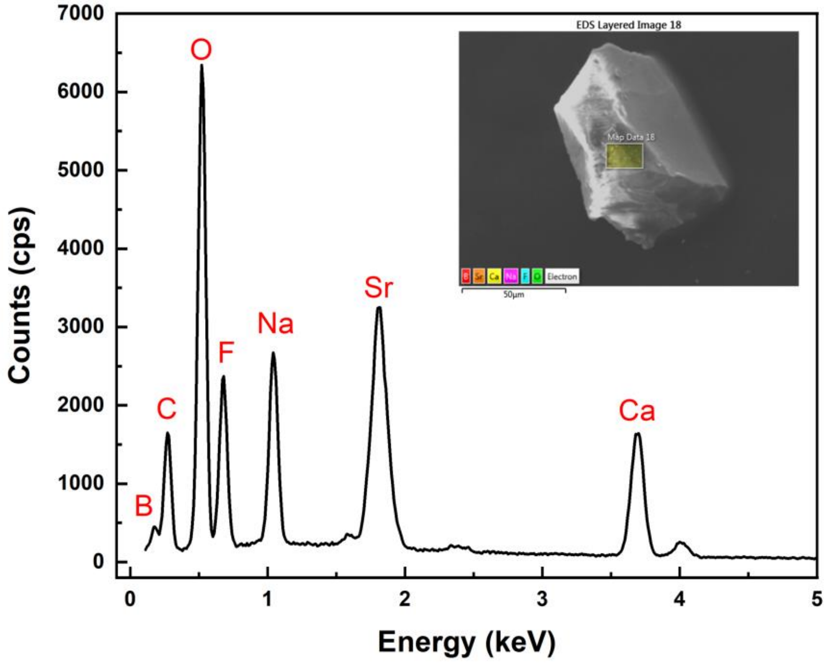

Figure S6. Energy dispersive X-ray spectrum of $Li_2Na_4Ca_7Sr_2B_{13}O_{27}F_9$. The results confirm the existence of F. Besides, the band corresponding to C originates from dimethicone, which was used to stabilize the crystal in the silicon dioxide slide.

Note: The final R indexes of $LiNa_{11}B_{28}O_{48}$ are $R_1 = 0.0625$ and $wR_2 = 0.1347$, it is larger than the general value. The situation has shown little improvement despite several attempts of adjusting experiments, picking crystals, collecting data, and analyzing structure. This may attribute to the weak diffraction and complex structure of $LiNa_{11}B_{28}O_{48}$ crystal, which also explains why ALERT level B is flagged in the checkCIF file for this compound.
